# Methyl-dependent auto-regulation of the DNA *N*^6^-adenine methyltransferase AMT1 in the unicellular eukaryote *Tetrahymena thermophila*

**DOI:** 10.1093/nar/gkaf022

**Published:** 2025-01-24

**Authors:** Lili Duan, Haicheng Li, Aili Ju, Zhe Zhang, Junhua Niu, Yumiao Zhang, Jinghan Diao, Yongqiang Liu, Ni Song, Honggang Ma, Kensuke Kataoka, Shan Gao, Yuanyuan Wang

**Affiliations:** MOE Key Laboratory of Evolution & Marine Biodiversity and Institute of Evolution & Marine Biodiversity, Ocean University of China, Qingdao 266003, China; Laboratory for Marine Biology and Biotechnology, Laoshan Laboratory, Qingdao 266237, China; Division of Chromatin Regulation, National Institute for Basic Biology, Okazaki 444-8585, Japan; MOE Key Laboratory of Evolution & Marine Biodiversity and Institute of Evolution & Marine Biodiversity, Ocean University of China, Qingdao 266003, China; Laboratory for Marine Biology and Biotechnology, Laoshan Laboratory, Qingdao 266237, China; MOE Key Laboratory of Evolution & Marine Biodiversity and Institute of Evolution & Marine Biodiversity, Ocean University of China, Qingdao 266003, China; Laboratory for Marine Biology and Biotechnology, Laoshan Laboratory, Qingdao 266237, China; MOE Key Laboratory of Evolution & Marine Biodiversity and Institute of Evolution & Marine Biodiversity, Ocean University of China, Qingdao 266003, China; Laboratory for Marine Biology and Biotechnology, Laoshan Laboratory, Qingdao 266237, China; MOE Key Laboratory of Evolution & Marine Biodiversity and Institute of Evolution & Marine Biodiversity, Ocean University of China, Qingdao 266003, China; Laboratory for Marine Biology and Biotechnology, Laoshan Laboratory, Qingdao 266237, China; MOE Key Laboratory of Evolution & Marine Biodiversity and Institute of Evolution & Marine Biodiversity, Ocean University of China, Qingdao 266003, China; Laboratory for Marine Biology and Biotechnology, Laoshan Laboratory, Qingdao 266237, China; MOE Key Laboratory of Evolution & Marine Biodiversity and Institute of Evolution & Marine Biodiversity, Ocean University of China, Qingdao 266003, China; Laboratory for Marine Biology and Biotechnology, Laoshan Laboratory, Qingdao 266237, China; MOE Key Laboratory of Evolution & Marine Biodiversity and Institute of Evolution & Marine Biodiversity, Ocean University of China, Qingdao 266003, China; Laboratory for Marine Biology and Biotechnology, Laoshan Laboratory, Qingdao 266237, China; Key Laboratory of Marine Medicine, Chinese Ministry of Education, School of Medicine and Pharmacy, Ocean University of China, Qingdao 266003, China; MOE Key Laboratory of Evolution & Marine Biodiversity and Institute of Evolution & Marine Biodiversity, Ocean University of China, Qingdao 266003, China; Laboratory for Marine Biology and Biotechnology, Laoshan Laboratory, Qingdao 266237, China; Division of Chromatin Regulation, National Institute for Basic Biology, Okazaki 444-8585, Japan; Basic Biology Program, Graduate Institute for Advanced Studies, The Graduate University for Advanced Studies, SOKENDAI, Okazaki 444-8585, Japan; MOE Key Laboratory of Evolution & Marine Biodiversity and Institute of Evolution & Marine Biodiversity, Ocean University of China, Qingdao 266003, China; Laboratory for Marine Biology and Biotechnology, Laoshan Laboratory, Qingdao 266237, China; MOE Key Laboratory of Evolution & Marine Biodiversity and Institute of Evolution & Marine Biodiversity, Ocean University of China, Qingdao 266003, China; Laboratory for Marine Biology and Biotechnology, Laoshan Laboratory, Qingdao 266237, China

## Abstract

DNA *N*^6^-methyladenine (6mA) is a potential epigenetic mark involved in gene transcription in eukaryotes, yet the regulatory mechanism governing its methyltransferase (MTase) activity remains obscure. Here, we exploited the 6mA MTase AMT1 to elucidate its auto-regulation in the unicellular eukaryote *Tetrahymena thermophila*. The detailed endogenous localization of AMT1 in vegetative and sexual stages revealed a correlation between the 6mA reestablishment in the new MAC and the occurrence of zygotically expressed AMT1. Catalytically inactive AMT1 reduced 6mA level on the *AMT1* gene and its expression, suggesting that AMT1 modulated its own transcription via 6mA. Furthermore, AMT1-dependent 6mA regulated the transcription of its target genes, thereby affecting cell fitness. Our findings unveil a positive feedback loop of transcriptional activation on the *AMT1* gene and highlight the crucial role of AMT1-dependent 6mA in gene transcription.

## Graphical Abstract

**Graphical Abstract:**
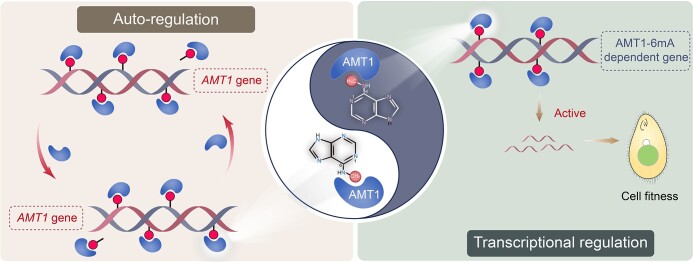

## Introduction

DNA *N*^6^-methyladenine (6mA), as a potential eukaryotic epigenetic mark, is reported to be involved in transcriptional regulation in eukaryotes [1–15]. However, the relationship between 6mA and transcription varies across the tree of life. Depending on the organism, 6mA may either activate or repress transcription. For instance, 6mA is associated with active transcription in the ciliate *Tetrahymena thermophila* (hereafter referred to as *Tetrahymena*) [10, 11], the unicellular alga *Chlamydomonas reinhardtii* [2], the nematode *Caenorhabditis elegans* [4], early-diverging fungi [16], and the vascular plant *Arabidopsis thaliana* [5]. In contrast, 6mA generally represses gene transcription in the lepidopteran *Bombyx mori* [17], the African clawed frog *Xenopus laevis* [18], the zebrafish *Danio rerio* [15], and various mammalian cells (pig, mouse, and human) [12, 13, 15, 19]. This divergence is most likely due to differences in their 6mA methyltransferases (MTases). 6mA MTases have been identified in several eukaryotes, including N6AMT1 in human, DAMT-1 in *C. elegans*, BmMETTL4 in *B. mori* [17], METTL4 in mammalian mitochondrion [20], AMT1 in *Tetrahymena* [11], and MTA1 in the ciliate *Oxytricha trifallax* (hereafter referred to as *Oxytricha*) [21]. In *C. elegans*, *B. mori*, and mammalian mitochondrion, their 6mA MTases, belonging to the METTL4 subclade of the MT-A70 family [22], are associated with low 6mA levels, non-consensus motifs, and non-genic regions [4, 17, 20]. In contrast, 6mA MTases in *Tetrahymena* and *Oxytricha*, categorized as the AMT1 subclade of the MT-A70 family, are related with abundant 6mA, ApT motif, and Pol II transcription [11, 21].

*Tetrahymena* possesses two types of nuclei, the transcriptionally active macronucleus (MAC) and the transcriptionally inert micronucleus (MIC) [23–25]. 6mA is the only detectable DNA methylation in *Tetrahymena*, specifically in the ApT motif [3, 10, 26], making it an ideal system for studying the function and regulation of 6mA. In the vegetative stage, 6mA is exclusively present in the MAC, but not in the MIC [11]. During the sexual stage (conjugation), the zygotic MIC differentiates into the new MACs for the sexual progeny [27], whereas 6mA in the developing new MAC must be re-established from the unmethylated germline MIC [11]. We identified the major 6mA MTase AMT1 in *Tetrahymena* [11]. Deletion of *AMT1* is not lethal, but it severely impairs cell growth [11]. The abnormally large contractile vacuole in Δ*AMT1* cells is associated with reduced expression of the *RAB46* gene, which encodes a Rab family GTPase involved in membrane trafficking and is regulated by AMT1-catalyzed 6mA [11]. However, how AMT1 is regulated and how it regulates transcription remain unknown.

In this study, we analyzed the cellular localization of AMT1 generated from different sources in *Tetrahymena*, including the maternal MAC (maternal AMT1) and the new MAC (zygotic AMT1). We demonstrated that the reestablishment of 6mA in the new MAC correlated with the expression of zygotic AMT1. Using a catalytically inactivated AMT1, we discovered that AMT1 modulated its own transcription by adjusting 6mA level on its coding gene. By comparing the transcription profile across different AMT1 conditions, including AMT1 deletion, mutation, and dosage manipulation, we found that AMT1-dependent 6mA regulated the transcription of its targeted genes, thus affecting the cell fitness of *Tetrahymena*.

## Materials and methods

### Cell culture

Wild-type (WT) SB210 and CU428 strains of *T. thermophila* were obtained from the *Tetrahymena* Stock Center (http://tetrahymena.vet.cornell.edu). Cells were cultured to the log phase (2–3 × 10^5^ cells/ml) in SPP medium at 30°C [28]. For mating, cells in the log phase were starved in 10 mM Tris–HCl, pH 7.4 for 16–18 h at 30°C. Mating was initiated by mixing cells expressing different mating types [29, 30]. All strains were generated using the somatic and germline transformation with standard procedures [31, 32], and detailed information was described in the Supplementary Materials and Methods. The primers used for generating the constructs are listed in Supplementary Table S1, and all strains are provided in Supplementary Table S2.

### Phenotypic analyses

For growth analyses, cells at the density of 0.8 × 10^5^ cells/ml were inoculated in 10 ml of SPP medium and incubated at 30°C. The cell concentration was measured at the indicated timepoints after inoculation by Coulter Counter Z2 (Beckman). Statistical analysis was performed as described [33].

### Immunofluorescence staining

Immunofluorescence (IF) staining was performed as previously described [11]. The primary antibodies were anti-6mA antibody (rabbit, 1:2000, Synaptic Systems, 202003) and anti-HA antibody (rabbit, 1:200, Cell Signaling, 3724), and the secondary antibodies were Goat anti-Rabbit IgG (H + L)–Alexa Fluor 555 (1:4000, Invitrogen, A-21428) and Goat anti-Mouse IgG (H + L)–Alexa Fluor 488 (1:4000, Invitrogen, A11001). For the co-staining, cells were incubated with anti-6mA antibody and anti-HA antibody (mouse, 1:500, Covance, MMS-101P), respectively. Zeiss Axio Imager Z2 and Olympus BX43 microscope were used for imaging.

### UHPLC–QQQ-MS/MS analysis

Genomic DNA was extracted by phenol–chloroform extraction as described previously [2]. The DNA (500 ng) was digested into mononucleotides by incubating with DNase I (1 U, NEB, M0303L), Fast AP (1 U, Invitrogen, EF0651), and snake venom phosphodiesterase I (0.005 U, Sigma, P4506) at 37°C for 17 h. The molecular masses of the mononucleotides were analyzed by ultra-high-performance liquid chromatography–tandem mass spectrometry (UHPLC–QQQ-MS/MS) as described previously [10]. The selective multiple reaction monitoring transitions were detected under *m/z* 266/150 for 6mA and *m/z* 252/136 for dA. The ratio of 6mA/A was quantified by the calibration curves from nucleotide standards running simultaneously.

### Western blot

After trichloroacetic acid treatment, cells were lysed in the sodium dodecyl sulfate sample buffer as described previously [34] and subjected to western blotting. Proteins on the blot were detected by anti-HA antibody (rabbit, 1:2000, Cell Signaling, 3724) or anti-alpha-tubulin antibody (mouse, 1:8000, Sigma, T6199), followed by the incubation with the secondary antibodies Goat anti-Rabbit IgG (H + L) HRP Conjugate (1:8000, TransGen Biotech, HS101-01) and Goat anti-Mouse IgG (H + L) HRP Conjugate (1:8000, TransGen Biotech, HS201-01), respectively.

### Quantitative PCR of 6mA IP samples

6mA immunoprecipitation (6mA IP) was performed as previously described [2], and detailed procedures were described in Supplementary Materials and Methods. For 6mA IP samples, the 6mA enrichment of representative 6mA-methylated sites identified by single-molecule, real-time circular consensus sequencing (SMRT-CCS) was analyzed by quantitative polymerase chain reaction (qPCR) using primers indicated in Supplementary Table S1. Fold enrichment was calculated using the input DNA as the reference. The degree of 6mA enrichment was calculated by comparing Ct values of methylated genes with that of unmethylated rDNA. Three technical replicates were performed and averaged to represent the relative abundance. The equation utilized was as follows: ΔCt = Ct(IP sample) − Ct(Input sample); ΔΔCt = ΔCt(methylated gene) − ΔCt(unmethylated rDNA).

### RNA sequencing

Total RNA was extracted from 1 × 10^6^ cells of vegetatively growing WT and AMT1-RNAi cells treated with 1 μg/ml Cd^2+^ for 17 h, as well as untreated AMT1-APPA, Δ*AMT1*, and WT cells, using the FastPure Cell/Tissue Total RNA Isolation Kit-Box 2 (Vazyme, RC101-01). AMT1-APPA and Δ*AMT1* RNA samples were prepared separately, with WT RNA samples (WT^a^ for AMT1-APPA and WT^b^ for Δ*AMT1*) extracted simultaneously as the internal controls for each set. The extracted RNA was then mixed with an equal amount of zebrafish RNA as a spike-in. Sequencing libraries were generated using NEBNext Ultra RNA Library Prep Kit for Illumina (NEB, USA, E7530L) following the manufacturer's recommendations. Index codes were added to the attribute sequences for each sample. RNA sequencing (RNA-seq) was performed by NovaSeq Xplus PE150. Trim Galore-0.6.7 was used to remove sequencing adapters and obtain high-quality reads (length >36 and quality >20) [35]. Retained reads were mapped to the *Tetrahymena* MAC reference genome using Hisat2-2.1.0 [36, 37]. Reads with multiple mapping in the mapping SAM file were deleted. After removing PCR duplicates using Picard MarkDuplicates-2.18.29 (http://broadinstitute.github.io/picard/), the retained reads were counted by FeatureCounts-1.6.1 [38]. To correct for sequencing depth, the raw data were additionally mapped to the zebrafish genome (NCBI RefSeq assembly: GCF_000002035.6), and PCR duplicates were removed. For each sample, the natural logarithm of the reads mapped to the zebrafish genome was calculated, and the average of these values was determined. The number of zebrafish genome reads for each sample was divided by the exponential of this average to obtain size factors. These size factors were then applied in the R package DESeq2 to normalize sequencing depth and perform differential expressed gene analysis. Gene Ontology (GO) analysis was performed by TBtools-II [39].

### RT-qPCR

Total RNAs were reverse-transcribed using a HiScript III 1st Strand cDNA Synthesis Kit with random hexamer (Vazyme, R312-02). Reverse transcriptase (RT)-qPCR was performed using EvaGreen Express 2× qPCR MasterMix-Low ROX (Abm, MasterMix-LR) in 7500 Fast Real-Time PCR System as described previously [11]. Two different pairs of primers were used for RT-qPCR of *AMT1* (AMT1-RTqPCR-f4905/AMT1-RTqPCR-r5155) and *RAB46* (RAB46-RTqPCR-f2018/RAB46-RTqPCR-r2246). *JMJ1* (TTHERM_00185640, JMJ1-RTqPCR-f2244/JMJ1-RTqPCR-r3176) were used as the internal control. Messenger RNA (mRNA) levels of endogenous *AMT1* were measured using the primers HA-RT-f and AMT1-RT-r. mRNA levels of ectopically expressed *AMT1* were detected using the primers 3× G196-RT-f and AMT1-RT-r. All qPCR primers were listed in Supplementary Table S1. Three technical replicates were performed and averaged to represent the relative abundance. The equation utilized was as follows: ΔCt = Ct(targeted gene) − Ct(internal control-JMJ1); ΔΔCt = ΔCt(targeted strain) − ΔCt (control strain).

### Sample preparation and data analysis of SMRT-CCS

Genomic DNA was extracted from AMT1-APPA, and AMT1-RNAi cells treated with 1 μg/ml Cd^2+^ treatment for 17 h, using Wizard^®^ Genomic DNA Purification Kit (Promega, A1120). Genomic gDNA were sheared to 3–5 kb length by the Bioruptor (Diagenode SA, UCD-300I), before used to generate sequencing libraries for the PacBio Sequel II System.

SMRT sequencing data were analyzed following previously described procedures [9, 40]. The subreads were sorted using samtools, and the ccs module was applied to generate CCS and sequencing information for each single molecule. Custom Perl scripts were employed to aggregate sequencing information and extracted high-confidence single molecules (passes ≥20×) for subsequent analysis. These molecules were aligned to their consensus sequences using BLASR. Inter-pulse duration (IPD, referring to the time interval between the incorporation of nucleotides during DNA polymerization) ratios were computed using ipdSummary (SMRT Link v11.0, Pacific Biosciences). Molecules exhibiting global dispersion, defined as a standard deviation (SD) of IPD ratios ≥0.35 on the Watson and/or Crick strands for all unmethylated adenine sites (IPD ratios <2.8) within each molecule, were excluded. Molecules demonstrating local dispersion of IPD ratios with high-density N* sites were also removed to minimize the interference of sequencing instability. High-density N* sites were defined as non-A bases with IPD ratios ≥2.8, occurring within a distance of ≤25 bp and with a count of ≥4 on the same strand. CCS of selected single molecules were mapped back to the *Tetrahymena* MAC genome [37] using Blastn with the parameters “-max_hsps 1, -max_target_seqs 1.” MAC-mapped single molecules were selected for further analysis. The threshold for 6mA IPD ratios across all single molecules was determined through deconvolution of the bimodal distribution of IPD ratios. The methylation penetrance of an ApT site was defined as the ratio of methylated adenines to the total number of adenines at a specific site across all sequencing reads. The methylation level of a gene was represented by Σ*P*, which was the summation of the penetrance values of all ApT sites within that gene. Adenine sites with methylation supported by at least three individual DNA molecules were classified as high-confidence 6mA sites.

### Data analysis for chromatin immunoprecipitation sequencing and micrococcal nuclease sequencing

The chromatin immunoprecipitation sequencing (ChIP-seq) and micrococcal nuclease sequencing (MNase-seq) data were obtained from our previous work [11, 37]. The raw sequencing data were subjected to quality control using Trim Galore to select out low-quality reads. Subsequently, Hisat2 was employed to align the reads to the *Tetrahymena* MAC reference genome [37] with the parameters “- -no-mixed, - -no-discordant, - -no-spliced-alignment.” PCR duplicates were removed using Picard MarkDuplicates. A custom script was utilized to select fragments with lengths ranging from 120 to 260 bp. For ChIP-seq analysis, the ChIP enrichment ratio for each gene was calculated by dividing the normalized number of fragments mapped to the gene in the ChIP sample by that in the input sample. In the case of MNase-seq data, the resulting BAM files were converted to BED format using BEDTools [41]. Nucleosome positions were determined using the NucPosSimulator [42]. The positioning degree of each nucleosome was calculated as the number of fragments within 25 bp upstream and downstream of the nucleosome dyad, divided by the number of fragments within 75 bp upstream and downstream of the nucleosome dyad [43]. The nucleosome positioning degree for each gene was defined as the average positioning degree of the two or three nucleosomes immediately adjacent to the transcription start site (TSS).

## Results

### The correlation between 6mA reestablishment and zygotically expressed AMT1

**Figure 1.**
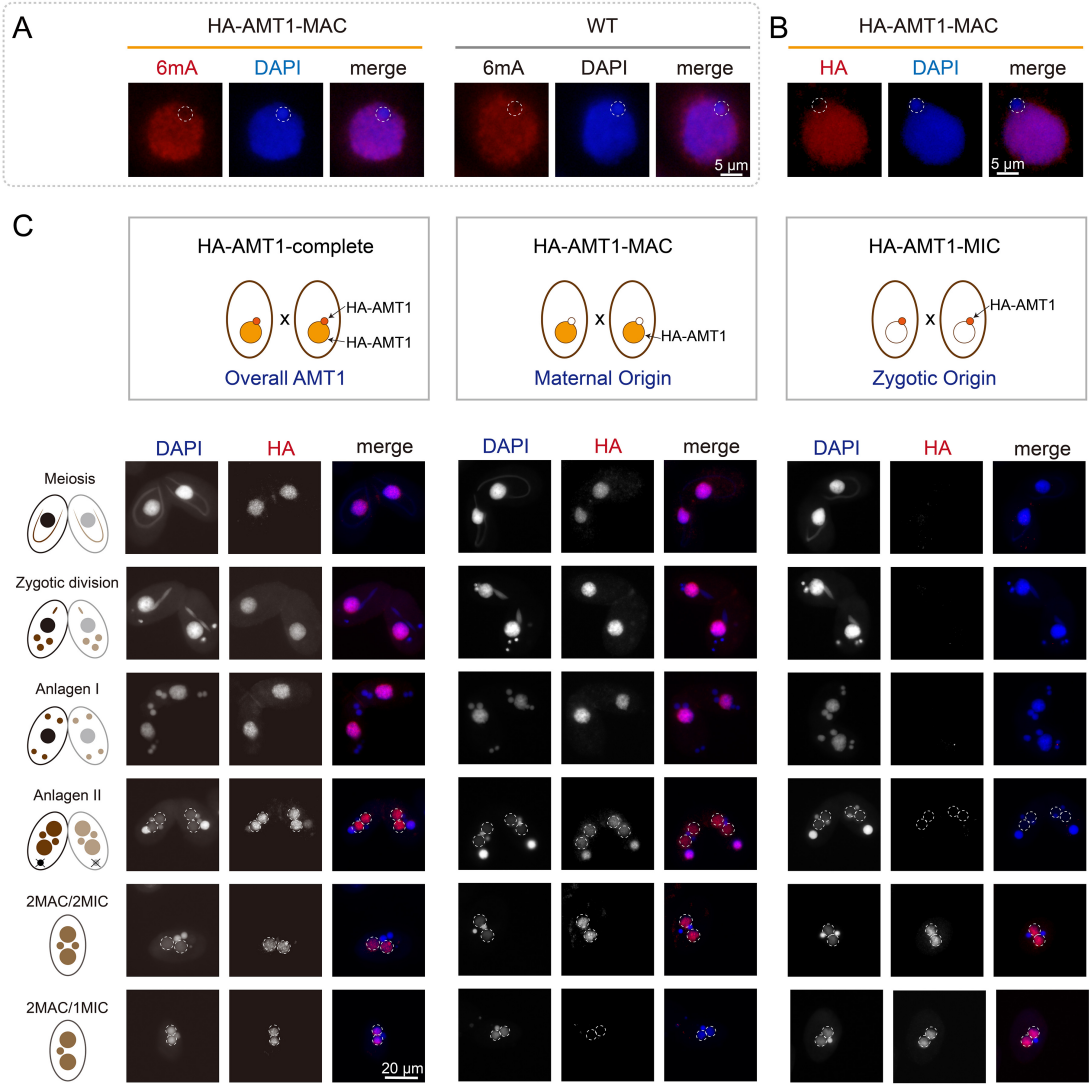
Cellular localization of AMT1. (**A**) IF staining using anti-6mA antibody in HA-AMT1-MAC and WT cells. DNA was stained with DAPI. MIC was encircled by dotted line. (**B**) IF staining of HA-tagged AMT1 in somatic HA-AMT1-MAC cells. AMT1 (HA) was absent in the MIC (dotted circles). (**C**) IF staining of HA-tagged AMT1 in HA-AMT1-complete (MAC: AMT1 tagged by HA; MIC: tagged by HA), HA-AMT1-MAC (MAC: tagged by HA; MIC: WT), and HA-AMT1-MIC (MAC: WT; MIC: tagged by HA) cells during conjugation. Maternal AMT1 persisted until the 2MAC/2MIC stage and then rapidly diminished during the 2MAC/1MIC stage, as demonstrated in HA-AMT1-MAC cells. Zygotic AMT1 was absent in both the maternal MAC and the new MAC (dotted circles) at the Anlagen II stage, as shown in HA-AMT1-MIC cells. Zygotic AMT1 began to appear in the new MAC at the 2MAC/2MAC stage.

To investigate the regulatory mechanism of the 6mA MTase AMT1, we first analyzed its cellular localization in the vegetative cells. In our previous study, we examined the epitope-tagged AMT1 from an ectopic locus (OE-HA-AMT1) [11]. In this study, we generated cells expressing *N*-terminally hemagglutinin (HA)-tagged AMT1 protein from its endogenous locus in the MAC (Supplementary Fig. S1A and B, HA-AMT1-MAC). In HA-AMT1-MAC cells, 6mA levels were comparable to those in WT cells (Fig. 1A and Supplementary Fig. S1C), confirming that the HA-tag did not interfere with its methylation activity. Consistent with our previous observation in vegetative OE-HA-AMT1 cells [11], AMT1 localized exclusively in the MAC containing 6mA (Fig. 1B and Supplementary Fig. S1D).

**Figure 2.**
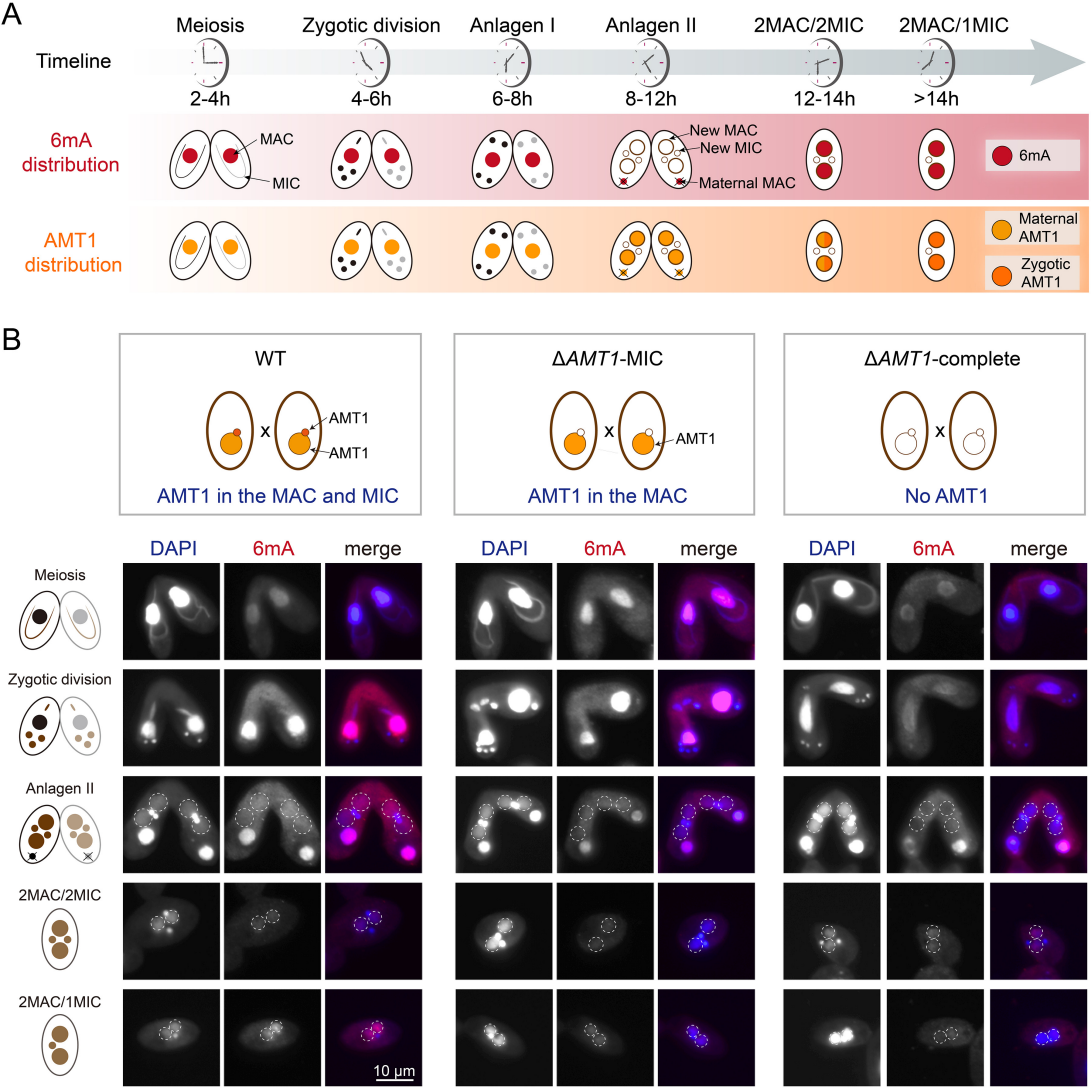
Cellular distribution of 6mA during conjugation. (**A**) Diagram showing the cellular localization of 6mA (top panel) and AMT1 (bottom panel) during conjugation. Nuclear events were used to ascertain conjugation stages. (**B**) IF staining of 6mA in WT (AMT1 was present in both MAC and MIC), Δ*AMT1*-MIC (AMT1 was deleted in the MIC), and Δ*AMT1*-complete (AMT1 was deleted in both MAC and MIC) cells during conjugation.

During conjugation, the zygotic MIC differentiates into the new MACs [44], in which 6mA MTase(s) should be recruited to deposit 6mA *de novo* [45, 46]. Using HA-AMT1-complete cells, which expressed HA-tagged AMT1 from both the maternal MAC and the new MAC, we observed that AMT1 initially localized in the maternal MAC and subsequently appeared in the new MAC soon after their enlargement (Fig. 1C, left panel). Intriguingly, 6mA was absent in the new MAC at this stage (Fig. 2A, top panel Anlagen II, Fig. 2B, left panel) and it occurred approximately 4 h after the new MAC formation (Fig. 2A, top panel 2MAC/2MIC, Fig. 2B, left panel) [11]. To explore whether this delay of 6mA deposition is due to the maternal or zygotic AMT1, we utilized strains expressing HA-tagged AMT1 either from the maternal MAC (maternal AMT1, HA-AMT1-MAC) or from the new MAC derived from zygotic MIC (zygotic AMT1, HA-AMT1-MIC) (Fig. 1C). Maternal AMT1 first localized in the maternal MAC during early conjugation and then in the new MAC lasting until the 2MAC/2MIC stage (Fig. 1C, middle panel). In contrast, zygotic AMT1 started to appear in the new MAC at 2MAC/2MIC stage (Fig. 1C, right panel), coinciding with the timing of the 6mA appearance.

To distinguish the respective contribution of maternal and zygotic AMT1, we analyzed 6mA levels by IF staining in WT, Δ*AMT1*-MIC (*AMT1* was deleted only in the MIC), and Δ*AMT1*-complete (*AMT1* was deleted in both MAC and MIC) cells (Fig. 2B). During early stages of conjugation, 6mA levels in the maternal MAC were significantly reduced in Δ*AMT1*-complete cells (Fig. 2B, right panel), but remained unchanged in Δ*AMT1*-MIC cells (Fig. 2B, middle panel), suggesting that 6mA levels in the maternal MAC are predominantly regulated by maternal AMT1. In contrast, in the new MAC of exconjugants (2MAC/2MIC and 2MAC/1MIC) of Δ*AMT1*-MIC and Δ*AMT1*-complete cells, both of which lacked zygotic AMT1, 6mA levels were lower compared to WT cells, strongly indicating that zygotic AMT1 is the main contributor to 6mA deposition in the new MAC. The residual 6mA levels observed in exconjugants imply the involvement of other potential 6mA MTase(s) [9, 11].

To investigate whether maternal AMT1 contributes to other events of conjugation, we conducted a side-by-side comparison of Δ*AMT1*-complete (without maternal AMT1) and Δ*AMT1*-MIC (with maternal AMT1) cells. Δ*AMT1*-complete cells exhibited relatively slower conjugation progression (Supplementary Fig. S1E) and much lower progeny survival rate (Supplementary Table S3) compared to Δ*AMT1*-MIC cells, clearly demonstrating the essential role of maternal AMT1 in conjugation.

### Auto-regulation of AMT1 by AMT1-catalyzed 6mA

**Figure 3.**
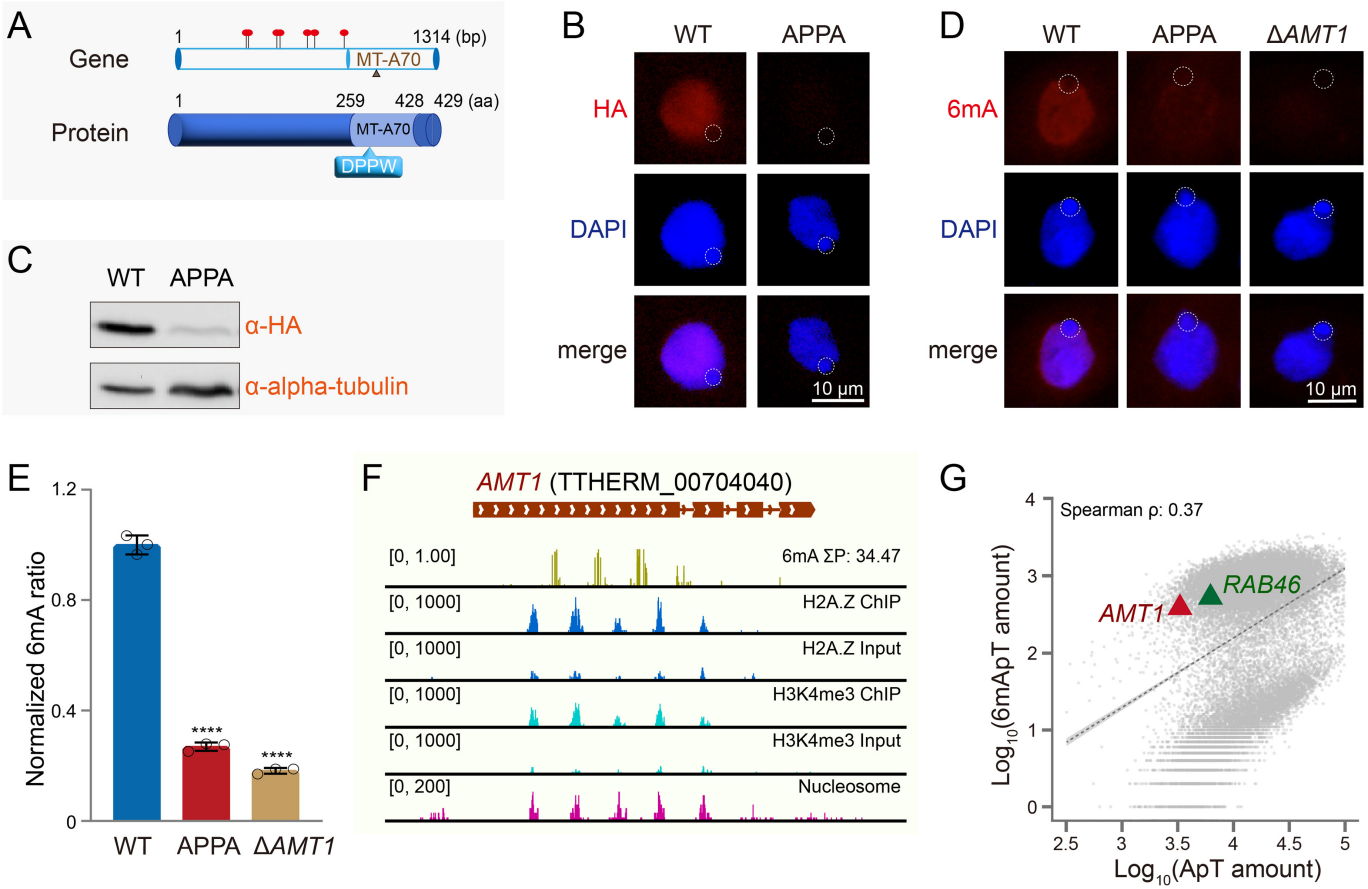
The reduction of *AMT1* expression in AMT1-APPA cells. (**A**) Illustration of the *AMT1* gene locus and its protein domain structure. (**B**) IF staining of HA-tagged AMT1 in HA-AMT1-MAC (WT) and HA-AMT1-APPA (APPA) cells. AMT1 (HA) was absent in the MIC (dotted circles). (**C**) Western blot of HA-tagged AMT1 in HA-AMT1-MAC (WT) and HA-AMT1-APPA (APPA) cells. Alpha-tubulin was used as the loading control. (**D**) IF staining of 6mA in WT, AMT1-APPA, and Δ*AMT1* cells. 6mA was absent in the MIC (dotted circles). (**E**) Mass spectrometry analysis of 6mA levels in WT, AMT1-APPA, and Δ*AMT1* cells. Data from three biological replicates were presented as histogram plots. Statistical analysis was performed using Student's *t*-test (****P < 0.0001). (**F**). Distributions of 6mA, H3K4me3, H2A.Z, and nucleosome in the *AMT1* gene locus (TTHERM_00704040) in WT cells. IGV (Integrative Genomics Viewer) snapshot tracks from top to bottom were as follows: gene model, 6mA levels, H2A.Z (ChIP and Input), H3K4me3 (ChIP and Input), and dyads of nucleosomes. (**G**). Scatterplot depicting the amount of ApT and 6mApT on all genes. Note that the 6mApT/ApT ratio on the *AMT1* and *RAB46* gene bodies (6mApT/ApT: 11.71% on *AMT1*, and 8.52% on *RAB46*) was higher than the average level (3.07%).

The MTase activity of AMT1 depends on the evolutionarily conserved DPPW motif within the MT-A70 domain (Fig. 3A) [11]. A D209A mutation in the catalytic DPPW motif of AMT1 nearly abolishes its MTase activity, as demonstrated by an *in vitro* study using a reconstituted AMT1 complex [47]. Additionally, substituting the DPPW motif with APPA in the *AMT1* gene reduced *AMT1* mRNA level, as demonstrated by our previous RT-qPCR analysis [11, 22]. To further analyze this downregulation, we generated cells expressing HA-tagged AMT1 carrying the APPA mutation (HA-AMT1-APPA) (Supplementary Fig. S2A and B). Complete replacement of the *AMT1* locus with the HA-tagged construct in the MAC was confirmed by Southern blot (Supplementary Fig. S2C). IF staining revealed a substantial reduction in AMT1 protein levels in HA-AMT1-APPA cells compared to HA-AMT1 cells (Fig. 3B and Supplementary Fig. S2D). This reduction was further corroborated by the western blot analysis (Fig. 3C). The decrease of *AMT1* mRNA levels was also confirmed by RT-qPCR (Supplementary Fig. S2E). These findings suggest that the APPA mutation impacts AMT1 at both mRNA and protein levels.

Consistent with its effects on AMT1, the APPA mutation reduced the global 6mA level in vegetative cells [11], as demonstrated by IF staining (Fig. 3D and Supplementary Fig. S2F) and 6mA mass spectrometry (Fig. 3E). This mutation also resulted in a phenotype similar to that observed in Δ*AMT1* cells, including slow cell growth and an abnormal contractile vacuole (Supplementary Fig. S2G) [11, 22]. To further explore the contribution of 6mA, we conducted a detailed investigation of 6mA sites within the *AMT1* gene. The chromatin environment of the *AMT1* gene in WT cells exhibited a conventional 6mA-related pattern [10], with 6mA sites relatively enriched at the 5′ end of the gene body (Fig. 3F and Supplementary Fig. S3A and B), between well-positioned nucleosomes (Fig. 3F and Supplementary Fig. S3B and C) decorated by abundant active transcriptional marks (H2A.Z and H3K4me3) (Fig. 3F and Supplementary Fig. S3B, D, and E) [10, 11]. Additionally, we found that the enrichment of 6mA sites on the *AMT1* gene body was higher (6mApT/ApT: 11.71%) than genes on average (3.07%) in WT cells (Fig. 3G). These findings suggest that the expression of *AMT1* might be modulated by 6mA.

**Figure 4.**
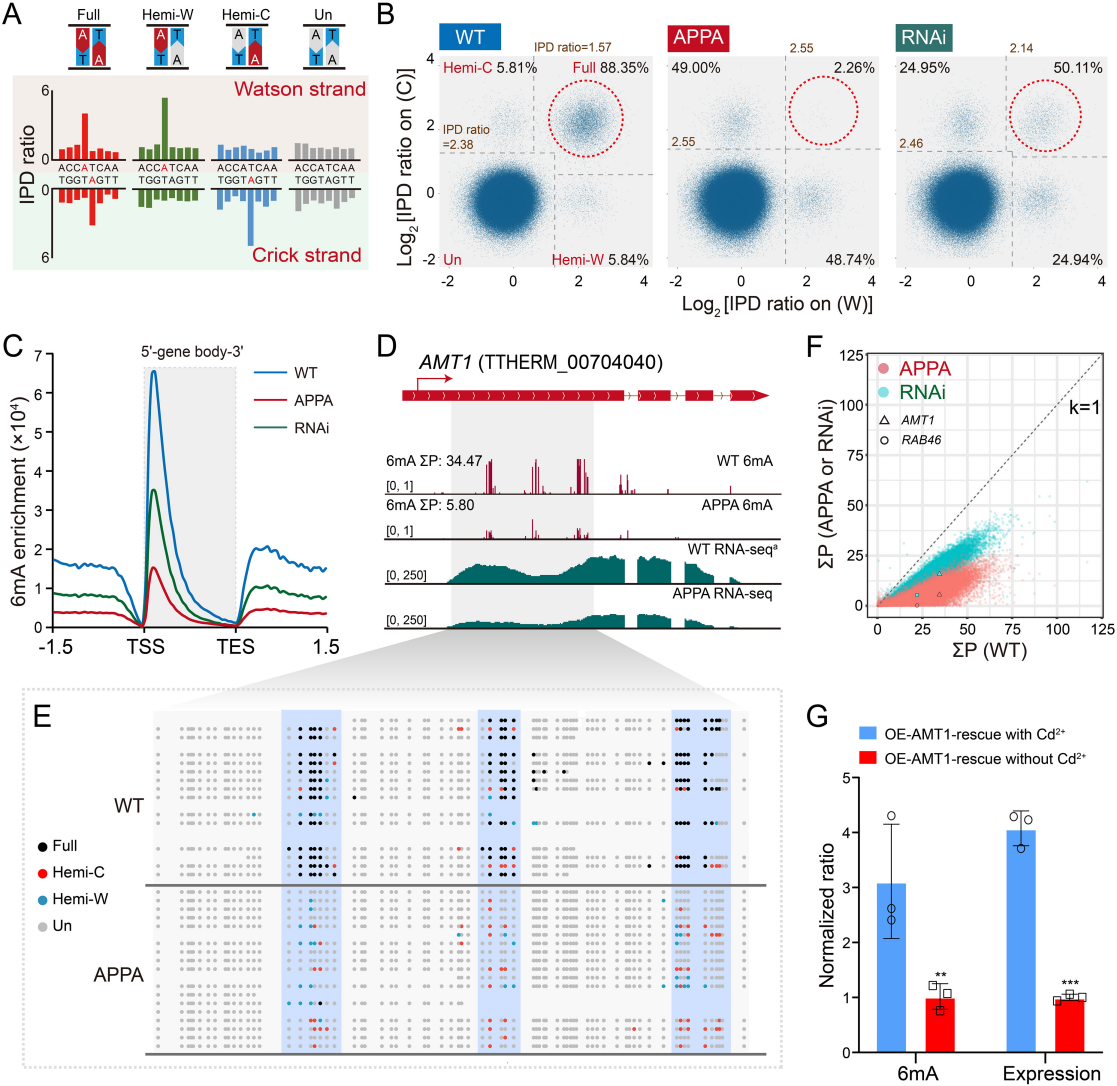
6mA distribution in AMT1-APPA and AMT1-RNAi cells. (**A**) Four states of ApT duplexes: full methylation (Full), 6mA methylation only on Watson strands (Hemi-W), 6mA methylation only on Crick strands (Hemi-C), and unmethylation (Un), distinguished by IPD ratios of adenine sites on Watson strand (W) and Crick strand (C), respectively. The IPD ratios and dispersion in typical single molecules were plotted for an 8-bp region containing one ApT site. (**B**) Demarcation of the four methylation states of ApT duplexes in WT (left), AMT1-APPA cells (middle), and AMT1-RNAi cells treated by 1 μg/ml Cd^2+^ for 17 h (right) by their IPD ratios on W and C strands, respectively. Note that WT cells contained abundant full methylation sites, which were nearly abolished in AMT1-APPA cells and decreased in AMT1-RNAi cells. The value represented the different IPD ratio cutoff. (**C**) Distribution of 6mA peaks around the gene body (TSS to TES) in WT, AMT1-APPA cells, and AMT1-RNAi cells treated by 1 μg/ml Cd^2+^ for 17 h. Genes were scaled to unit length and was extended to each side by unit length. One unit length was divided into 30 bins, and the degree of 6mA enrichment was calculated as the sum of penetrance within each bin. (**D**) IGV snapshot of the *AMT1* gene locus. Tracks from top to bottom were as follows: gene model, 6mA levels (SMRT-CCS data) of WT and AMT1-APPA cells, and mRNA levels (RNA-seq data) of WT^a^ (specifically for the transcription profile comparison with AMT1-APPA) and AMT1-APPA cells. (**E**) Single molecules covering the *AMT1* gene locus (highlighted in the IGV snapshot of Fig. 4D) in WT (top) and AMT1-APPA (bottom) cells. Note that in AMT1-APPA cells, full-6mApT on the *AMT1* gene was abolished, while the proportion of hemi-6mApT was increased compared to that in WT cells. (**F**) 6mA levels of individual genes were reduced in AMT1-APPA cells and AMT1-RNAi cells treated by 1 μg/ml Cd^2+^ for 17 h compared to WT cells. 6mA level of a specific gene was calculated as the sum of penetrance of all 6mApT positions on its gene body (Σ*P*). Note that the Σ*P* values for the *AMT1* and *RAB46* genes were located below the diagonal line (*k* = 1), indicating a greater reduction in their 6mA levels in AMT1-APPA and AMT1-RNAi cells. (**G**) qPCR analysis of 6mA IP samples and RT-qPCR of mRNA samples showed that both 6mA level and expression level of the endogenous *AMT1* gene were increased in OE-AMT1-rescue (HA-AMT1-APPA) cells induced with 1 μg/ml Cd^2+^ for 17 h, compared to cells without induction. Unmethylated rDNA was used as the internal control for qPCR, while *JMJ1* genes was used for RT-qPCR.

To address this possibility, we performed SMRT sequencing with the CCS mode to achieve the base resolution of 6mA sites for AMT1-APPA cells. We obtained 556,599 single molecules after stringent quality control, corresponding to 21.5× coverage of the *Tetrahymena* MAC genome (Supplementary Table S4). A total of 346,924 6mA sites were identified with high confidence (Supplementary Fig. S3F–H). The 6mA levels in AMT1-APPA cells were significantly reduced compared to WT cells, but were comparable to those in Δ*AMT1* cells (6mApT/ApT: 0.50% versus WT 2.03% versus Δ*AMT1* 0.53%) (Supplementary Table S5) [9], consistent with the IF staining and MS results (Fig. 3D and E and Supplementary Fig. S2F). A significantly lower full/hemi ratio (defined as the ratio between full-6mApT and hemi-6mApT) was also observed in AMT1-APPA cells (0.02 versus WT 7.58) (Fig. 4A and B and Supplementary Table S5), as previously reported in Δ*AMT1* cells (0.02) [9], highlighting the critical role of the DPPW motif for the MTase activity of AMT1 toward full methylation. Globally in AMT1-APPA cells, 6mA enrichment at the gene body was markedly decreased (Fig. 4C). Locally, the 6mA level on the *AMT1* gene was significantly reduced (6mA Σ*P*: 5.80 in AMT1-APPA versus 34.47 in WT) (Fig. 4D and E and Supplementary Fig. S3I), along with a corresponding decrease in *AMT1* mRNA level (Fig. 4D and Supplementary Fig. S2E). The decrease of 6mA level on *AMT1* was much greater than on most other 6mA-positive genes defined in WT cells (Fig. 4F).

To ascertain the auto-regulation of AMT1, we overexpressed the WT AMT1 from the ectopic *MTT1* locus in HA-AMT1-APPA cells (OE-AMT1-rescue) (Supplementary Fig. S4A and B). The expression level of the ectopic *AMT1* gene was significantly elevated following activation of the *MTT1* promoter by Cd^2+^ addition (Supplementary Fig. S4C). To further investigate this effect, we examined the endogenous *AMT1* gene by quantifying 6mA enrichments through qPCR in the 6mA IP samples, using primers specific to the endogenous *AMT1* gene (Supplementary Table S1). Our results demonstrated a substantial increase in 6mA enrichments on the endogenous *AMT1* gene (Fig. 4G, left panel), correlating with the observed rise in ectopic AMT1 levels (Supplementary Fig. S4C). This suggested that an increase in AMT1 protein could increase methylation level within the endogenous *AMT1* gene. Additionally, the increase in the 6mA level was accompanied by a corresponding enhancement in the mRNA level of the endogenous *AMT1* gene (Fig. 4G, right panel). These findings support the hypothesis that AMT1 enhances its own transcription by promoting the accumulation of 6mA on its gene body.

### AMT1-dependent 6mA in transcriptional regulation

The auto-regulation of AMT1 prompted us to explore its role in the transcriptional regulation of other genes. Previously, we identified *RAB46*, encoding a Rab family GTPase gene, as an AMT1-regulated gene crucial for contractile vacuole function in *Tetrahymena* [11]. The reduced expression of *RAB46* in Δ*AMT1* results in an abnormal large contractile vacuole [11]. In addition, 6mA level and mRNA level of *RAB46* in Δ*AMT1* showed a distinctly significant reduction compared to other genes (Supplementary Fig. S5A and B). The APPA mutation also led to the appearance of an abnormal contractile vacuole (Supplementary Fig. S2G). In line with our observation, the inactivation of AMT1 in AMT1-APPA cells led to a reduction in both 6mA and mRNA levels on the *RAB46* gene (Supplementary Fig. S5B and C), demonstrated, respectively, by SMRT-CCS and RNA-seq. This reduction was confirmed by qPCR analysis of 6mA IP samples and RT-qPCR analysis (Supplementary Figs S2E and S3I). These results suggest that the loss of AMT1 MTase activity leads to a reduction in 6mA level on the *RAB46* gene, thus impairing its transcription. Most genes showed a reduction in 6mA levels (Fig. 4F) as expected, with *AMT1* and *RAB46* exhibiting significant reductions, and *RAB46* showing a more pronounced decrease than *AMT1* (Fig. 4F and Supplementary Table S6). Intriguingly, only a subset of genes, including *AMT1* and *RAB46*, had downregulated mRNA levels, with *RAB46* showing a greater decrease compared to *AMT1* (Supplementary Table S6). The more substantial decrease in 6mA level of *RAB46* may contribute to its greater reduction in mRNA levels.

We then generated AMT1-RNAi cells capable of conditionally downregulating AMT1 through Cd^2+^ induction (Supplementary Fig. S6A and B). To optimize the Cd^2+^ induction, we treated AMT1-RNAi cells with various concentrations of Cd^2+^ (0, 1, 3, and 6 μg/ml). Our results showed that treatment with more than 1 μg/ml Cd^2+^ severely inhibited cell growth (Supplementary Fig. S6C, left panel). The growth of WT cells was slightly affected at 3 and 6 μg/ml CdCl_2_ (Supplementary Fig. S6C, right panel). This inhibitory effect was more pronounced in AMT1-RNAi cells, and those treated with more than 1 μg/ml CdCl_2_ were unable to propagate (Supplementary Fig. S6C), implying the additive contribution of RNAi to the effect CdCl_2_. Therefore, we used 1 μg/ml of Cd^2+^ for subsequent experiments.

**Figure 5.**
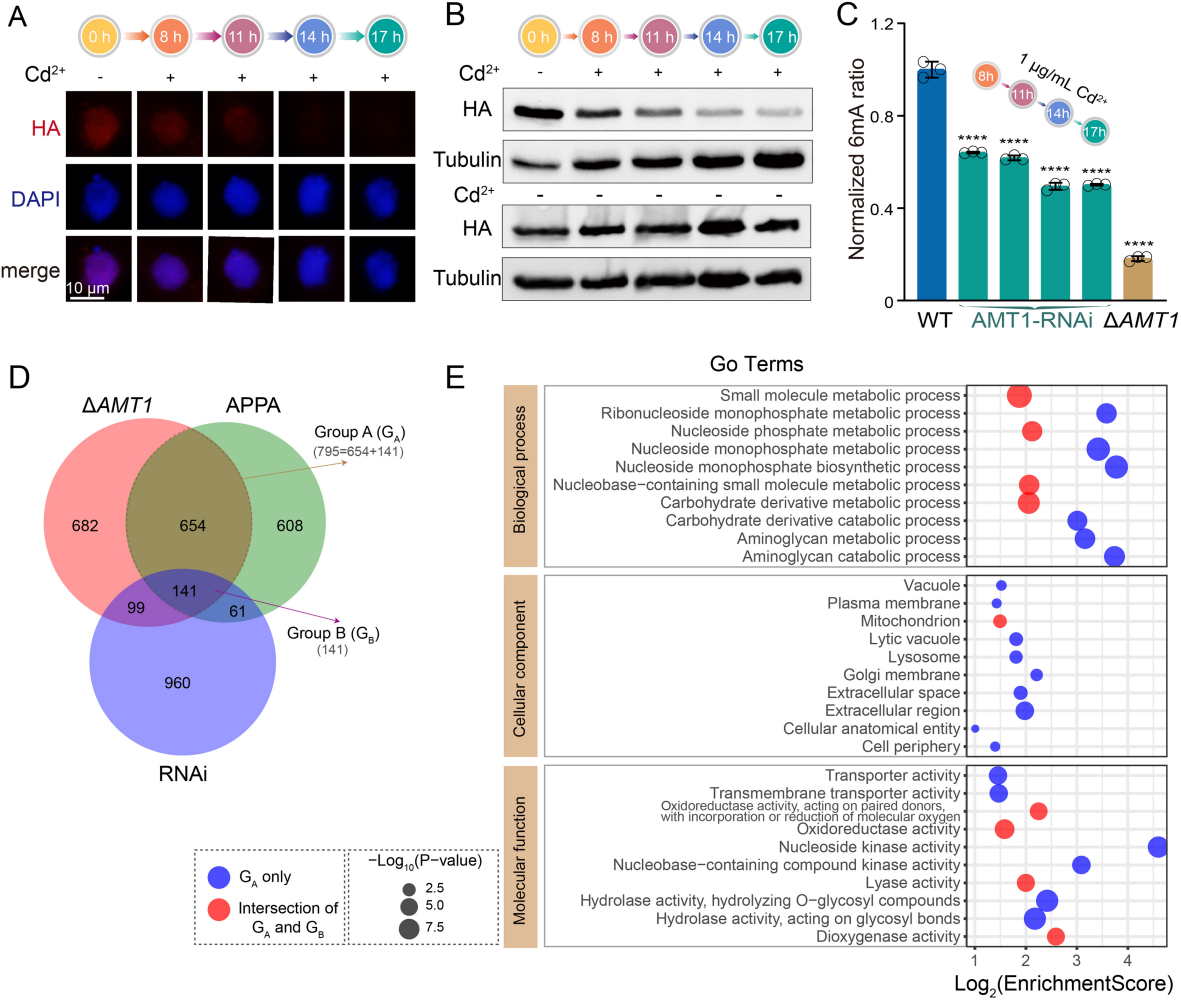
Gradual reduction of 6mA could be monitored by inducible AMT1-RNAi cells. (**A**) IF staining of HA-tagged AMT1 in AMT1-RNAi cells induced by 1 μg/ml Cd^2+^ for 0, 8, 11, 14, and 17 h. (**B**) Western blot of HA-tagged AMT1 in AMT1-RNAi cells induced by 1 μg/ml Cd^2+^ for 0, 8, 11, 14, and 17 h. AMT1-RNAi cells without cadmium induction (0 μg/ml Cd^2+^) were collected at the corresponding timepoints for comparison. Alpha-tubulin was used as the loading control. (**C**) Mass spectrometry analysis of 6mA levels in AMT1-RNAi cells induced by 1 μg/ml Cd^2+^ for 8, 11, 14, and 17 h. WT and Δ*AMT1* cells were included as the controls to demonstrate the significant differences in 6mA levels. Three biological replicates were used for each sample. Data were presented as histogram plots. Student's *t*-test was performed (****P< 0.0001). (**D**) Overlapping genes with reduced 6mA levels and decreased mRNA expressions (log_2_FoldChange ≤ −1, *P*_adj_ < 0.05) in Δ*AMT1*, AMT1-APPA, and AMT1-RNAi (induced with 1 μg/ml Cd^2+^ for 17 h) cells, compared to WT cells. A total of 795 genes (Group A) were downregulated in both AMT1-APPA and Δ*AMT1* cells. A total of 141 genes (Group B) exhibited downregulation among these three strains. (**E**) GO term analysis on 795 genes (Group A) that were co-downregulated in both 6mA and transcription levels in Δ*AMT1* and AMT1-APPA cells. G_A_ indicated pathways unique to Group A, while the intersection of G_A_ and G_B_ highlighted the intersected pathways between Group A and Group B. The top 10 terms with the lowest *P*-values in each GO class were displayed.

To monitor the AMT1 protein level, we introduced an *N*-terminal HA tag to the endogenous AMT1 in the AMT1-RNAi cells (Supplementary Fig. S6A and B). IF staining showed that the AMT1 protein level gradually decreased over time (8, 11, 14, and 17 h) in the MAC of Cd^2+^-induced AMT1-RNAi cells (Fig. 5A and Supplementary Fig. S6D). This gradual reduction was confirmed by western blot analysis (Fig. 5B), indicating that AMT1 protein level could be reduced in inducible AMT1-RNAi cells. MS analysis showed that 6mA levels gradually declined with the prolonged time course in Cd^2+^-treated AMT1-RNAi cells (Fig. 5C) but not in Cd^2+^-treated WT cells (Supplementary Fig. S6E). Given that 17 h of Cd^2+^ treatment dramatically reduced the global 6mA level (Fig. 5C), we performed SMRT-CCS to achieve the base resolution of 6mA sites for AMT1-RNAi cells, yielding 1,126,609 single molecules covering 24.5× of the *Tetrahymena* MAC genome (Supplementary Table S4). A total of 664,963 high-confidence 6mA sites were identified in AMT1-RNAi cells, revealing a significant reduction in 6mA levels compared to WT cells (Supplementary Fig. S7A–C and Supplementary Table S5, 6mApT/ApT: 1.02% versus WT 2.03%). Nonetheless, 6mA levels in AMT1-RNAi cells were higher than those in both Δ*AMT1* and AMT1-APPA cells (6mApT/ApT: Δ*AMT1* 0.53%, AMT1-APPA 0.50%) (Supplementary Table S5), likely due to the residual AMT1. A lower full/hemi ratio (Fig. 4B and Supplementary Table S5) and significantly decreased 6mA enrichment at the 5′ end of the gene body (Fig. 4C) were also observed in AMT1-RNAi cells. Notably, the decrease in 6mA level on the *AMT1* gene (Fig. 4F) further validated the auto-regulation of AMT1. Similarly, the 6mA level on the *RAB46* gene was reduced (Fig. 4F), suggesting that *RAB46* is indeed regulated by AMT1-dpendent 6mA.

Subsequently, we compared the transcriptomic profiles of AMT1-RNAi and WT cells treated with 1 μg/ml Cd^2+^ for 17 h, as well as AMT1-APPA, Δ*AMT1*, and untreated WT cells (Supplementary Fig. S8A). Considering that 6mA is associated with active transcription in *Tetrahymena* [11], we focused on downregulated genes (log_2_FoldChange < −1, *P*_adj_ < 0.05) that also exhibited reduced 6mA levels. We initially analyzed the downregulated genes between Δ*AMT1* and AMT1-APPA cells. A total of 795 genes were co-downregulated (Fig. 5D, Group A). GO analysis revealed their involvement in various biological pathways (Fig. 5E). Specifically, the downregulation of genes associated with metabolic pathways might explain the growth defects, while the large contractile vacuole phenotype could be linked to dysfunction in vacuole and transmembrane transporter-related processes. Additionally, we examined the overlap of downregulated genes with reduced 6mA levels among Δ*AMT1*, AMT1-APPA, and AMT1-RNAi cells (Fig. 5D, Group B). This analysis identified 141 co-downregulated genes (Supplementary Fig. S8B), which were involved in shared pathway related to molecular metabolic process and oxidoreductive biological processes (Fig. 5E and Supplementary Fig. S8B). These pathways may be particularly sensitive to changes in AMT1 levels and respond rapidly to the reduction of AMT1 expression. In conclusion, AMT1-dependent 6mA regulation likely impacts the transcription of these genes, which in turn affects the cell fitness of *Tetrahymena*.

The discrepancies in downregulated genes between the three cells could be attributed to variations in AMT1 and 6mA levels. The overlap between AMT1-APPA and Δ*AMT1* cells likely reflects their similarly low 6mA levels (6mApT/ApT: AMT1-APPA 0.50% versus Δ*AMT1* 0.53%). Differences between these two may be due to the compensatory activity from other 6mA MTase(s) in Δ*AMT1* cells, which was likely absent in AMT1-APPA cells. AMT1-RNAi cells showed a milder reduction in AMT1 level, resulting in a less pronounced decrease in 6mA levels (6mApT/ApT: AMT1-RNAi 1.02%), thus explaining its lower overlap of co-downregulated genes with other two cells.

## Discussion

**Figure 6.**
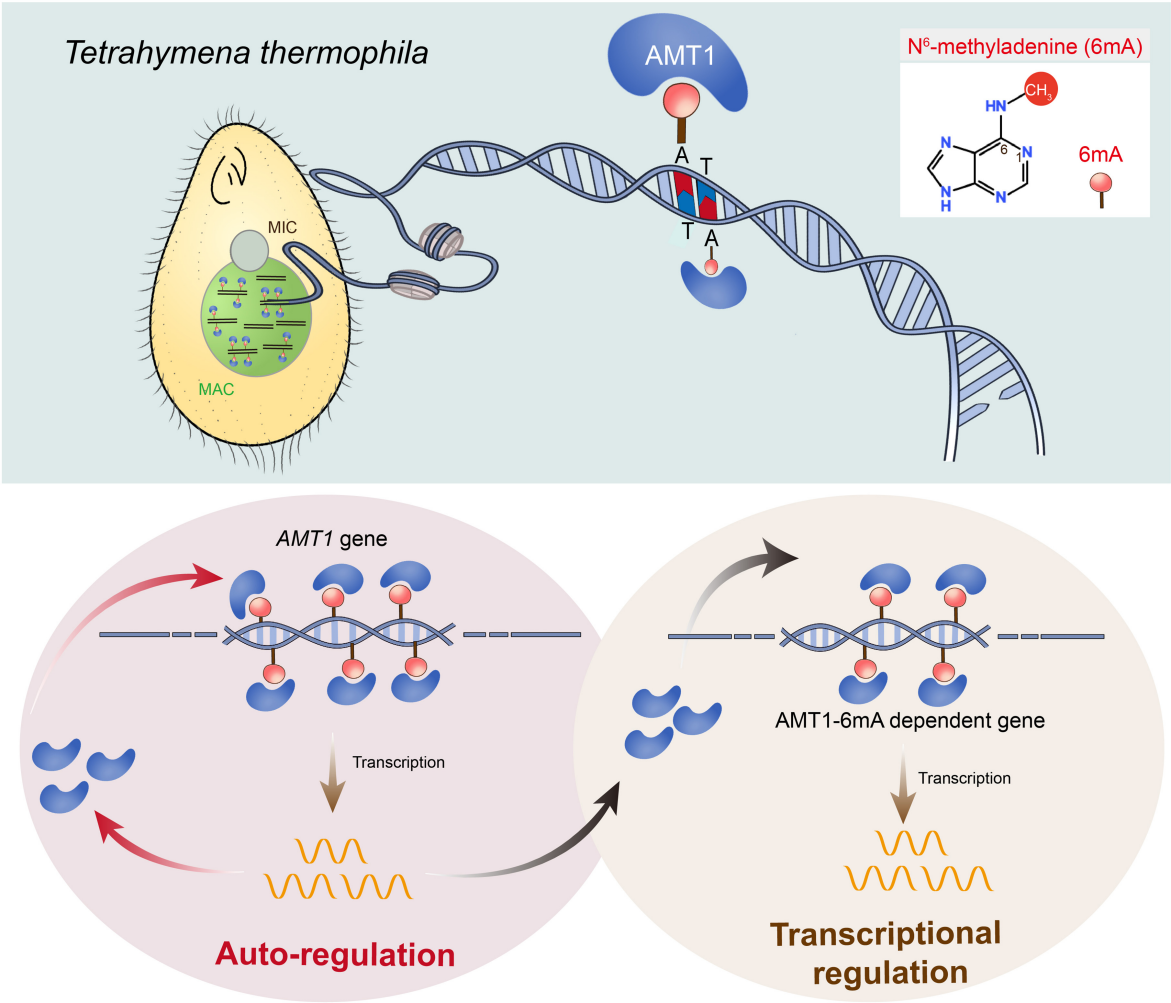
Diagram denoting AMT1 auto-regulation and transcriptional regulation mediated by AMT1-dependent 6mA. Top: MAC of *Tetrahymena thermophila* contains 6mA in the ApT motif catalyzed by AMT1. Bottom left: *AMT1* gene generates AMT1 protein, which then catalyzes 6mA on its own gene. Bottom right: AMT1 protein methylates other target genes, increasing their 6mA levels and thereby promoting their transcription.

As a novel DNA modification in eukaryotes, 6mA is reported to be regulated by its methyltransferases. However, the regulatory mechanism of 6mA MTase remains elusive. Here, we demonstrated both auto-regulation and transcriptional regulation of the 6mA MTase AMT1 in *Tetrahymena* (Fig. 6).

AMT1 was localized to the new MAC immediately after its formation, whereas 6mA appeared in the new MAC approximately 4 h later [10, 11]. In this study, we analyzed the localization of the AMT1 protein from different sources. We found that although maternal AMT1 was present in the new MAC at the stage of Anlagen II (the early stage of the new MAC formation), 6mA did not appear in the same nucleus. Instead, the timing of 6mA appearance correlated with that of zygotic AMT1 at the stage of 2MAC/2MIC, suggesting that maternal AMT1 in the early stage of the new MAC formation is not capable of depositing 6mA. Two scenarios could explain this disability. First, the catalytic activity of AMT1 may require cofactors that localize to the new MAC during the stage of 2MAC/2MIC. In vegetative cells, AMT1 (also known as MTA1) forms a complex with MTA9, p1, and p2, and its MTase activity is only detected when all three cofactors are present *in vitro* [21, 47, 48]. In the new MAC, the absence or lower concentration of these cofactors may limit the catalytic activity of AMT1. Second, since the preferred substrates of AMT1 are hemi-methylated ApT dinucleotides [9], unmethylated ApT dinucleotides in the early new MAC may not be methylated by AMT1 until hemi-methylated 6mApT is generated *de novo* by other 6mA MTases. Further research is needed to address these hypotheses.

During the vegetative stage, 6mA levels remain stable even after DNA replication [10, 11], likely due to the high activity of AMT1 [9]. The auto-regulation of AMT1 is an efficient way for achieving effective methylation through positive feedback, enhancing its activity by methylating its own gene. APPA mutation of *AMT1* abolished its MTase activity, leading to a decline in 6mA levels in the *AMT1* gene, which in turn downregulated *AMT1* transcription (Fig. 4D and E, Supplementary Figs S2E and S3I). Similarly, downregulation of AMT1 resulted in reduced 6mA levels in the *RAB46* gene, causing its declined expression (Supplementary Figs S2E, S3I, and S5B and C). These findings support the positive correlation between 6mA and transcription. However, the association between 6mA and global transcription was weak [10, 11]. We did not observe a clear correlation between the extent of 6mA decline and the degree of gene expression downregulation in Δ*AMT1*, AMT1-APPA, and AMT1-RNAi cells (Supplementary Fig. S8C). This could be attributed to the co-regulation of transcription by multiple factors, including 6mA and various histone marks (e.g. H2A.Z, H3K4me3). When 6mA levels decrease, these active histone marks may compensate to maintain the homeostasis of gene transcription. Alternatively, many Pol II-transcribed genes may have a threshold level of 6mA to sustain their expression.

6mA is typically abundant in early-diverging eukaryotes, including algae, basal fungi, and ciliates, and is associated with active transcription [2, 10, 11, 16, 21]. In the unicellular green alga *C. reinhardtii*, ∼0.4% of the genome is modified with 6mA, which exhibits a bimodal distribution around the TSS of active genes [2]. In basal fungi, 6mA levels can reach as high as 2.8%, with dense enrichment downstream of the TSS in actively transcribed genes [16]. Ciliates such as *Tetrahymena*, *Paramecium*, and *Oxytricha* also contain high levels of 6mA following the TSS [1, 6, 10, 11, 49]. Several potential mechanisms have been proposed to explain the association between 6mA and transcription [50]. The base conformation of 6mA destabilizes double-stranded DNA, facilitating the opening of transcriptional bubbles [51]. High levels of 6mA around TSS [10, 16] may slow down transcription elongation [52], with 6mA MTases potentially coupling with RNA polymerases to exploit this slow initiation phase. Additionally, 6mA is associated with well-positioned nucleosomes [10], which might prevent nucleosome sliding around the TSS, thus enhancing transcription. Chromatin marks correlated with 6mA, such as H2A.Z and H3K4me3 [11], are found in transcriptionally active genomic regions, further supporting the positive relationship between 6mA and transcription. Furthermore, 6mA reader proteins could recruit factors that promote transcription.

However, 6mA is relatively rare in the nuclear DNA of multicellular eukaryotes, including mouse and human, where 5-methylcytosine (5mC) prevails as the primary DNA methylation mark for gene repression [12,13]. The divergency between 5mC and 6mA may be closely associated with genome expansion and cell differentiation. Unicellular eukaryotes generally possess smaller genomes, necessitating the simultaneous transcription of a large proportion of their genome. In such cases, 6mA may serve as a default mechanism for transcriptional activation. In contrast, more complex multicellular eukaryotes with larger genomes often require transcriptional repression, resulting in a more dominant presence of 5mC and a gradual decrease in 6mA. This shift from predominantly active transcription marks to repressive marks reflects the evolutionary progression of gene expression regulation associated with increased cellular complexity and differentiation.

## Supplementary Material

gkaf022_Supplemental_Files

## Data Availability

The RNA-seq data generated in this study can be found under BioProject accession number PRJNA1193833. The processed ChIP-seq and MNase-seq files (bigWig format) and SMRT-CCS penetrance files are available on Zenodo (DOI:10.5281/zenodo.14604749) at https://doi.org/10.5281/zenodo.14604749.
