# Supplementary material for: Methyl-dependent auto-regulation of the DNA N6-adenine methyltransferase AMT1 in the unicellular eukaryote Tetrahymena thermophila
Source: Nucleic Acids Res. 2025 Jan 24;53(3):gkaf022. doi: 10.1093/nar/gkaf022 (PMC11760949; doi:10.1093/nar/gkaf022)
Supplement: gkaf022_Supplemental_Files [file gkaf022_supplemental_files.zip › Supplementary_0108_LHC.pdf]

Supplemental Figures 1-8 and Tables 1-6

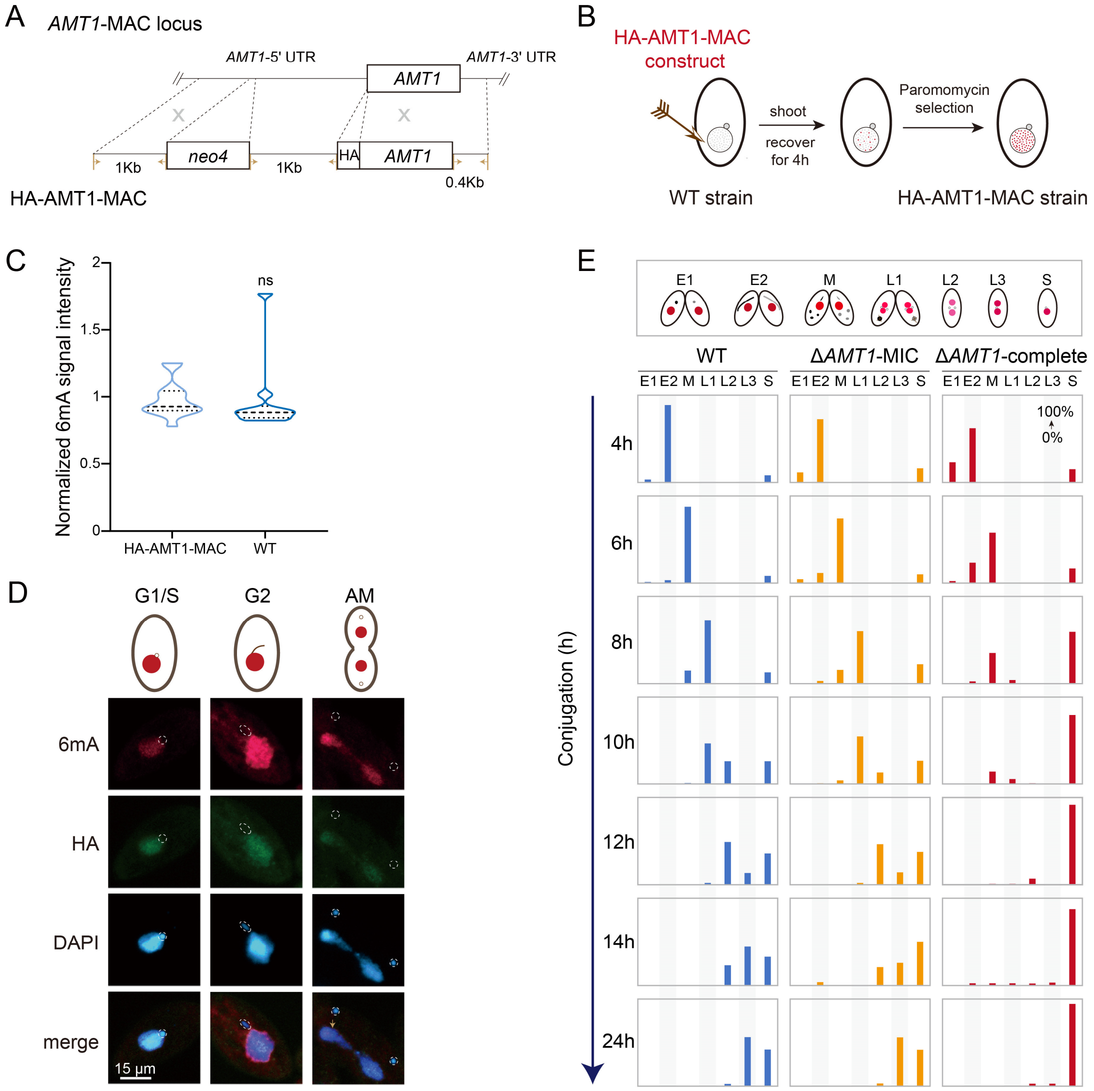

Fig. S1. Phenotypic analysis of HA-AMT1-MAC cells.

A. Schematic representation of the endogenous *AMT1* locus in the MAC of WT cells and the HA-AMT1-MAC construct.

B. Diagram illustrating the process of generating HA-AMT1-MAC cells. Starved WT cells were transformed with the HA-AMT1-MAC construct. True transformants were selected by increasing drug (paromomycin) pressure.

C. Statistical analysis of 6mA IF signal intensity in HA-AMT1-MAC and WT (SB210). Cell images were randomly selected (n (HA-AMT1-MAC) = 425 and n (WT) = 425) by ImageJ. Data are presented as violin plots using GraphPad Prism 8. Student's *t*-test was performed (ns > 0.05).

D. IF staining of HA-tagged AMT1 and 6mA in HA-AMT1-MAC cells. AMT1 (HA) and 6mA were absent in the MIC (dotted circles).

E. Conjugation progression analysis at different timepoints (4 h, 6 h, 8 h, 10 h, 12 h, 14 h and 24 h) based on their nuclear morphology: pre-meiosis (E1), meiosis (E2),

mitosis (M), and the three late stages of developed new MAC: late stage 1 (L1), late stage 2 (L2), late stage 3 (L3), and single cells (S). n > 200.

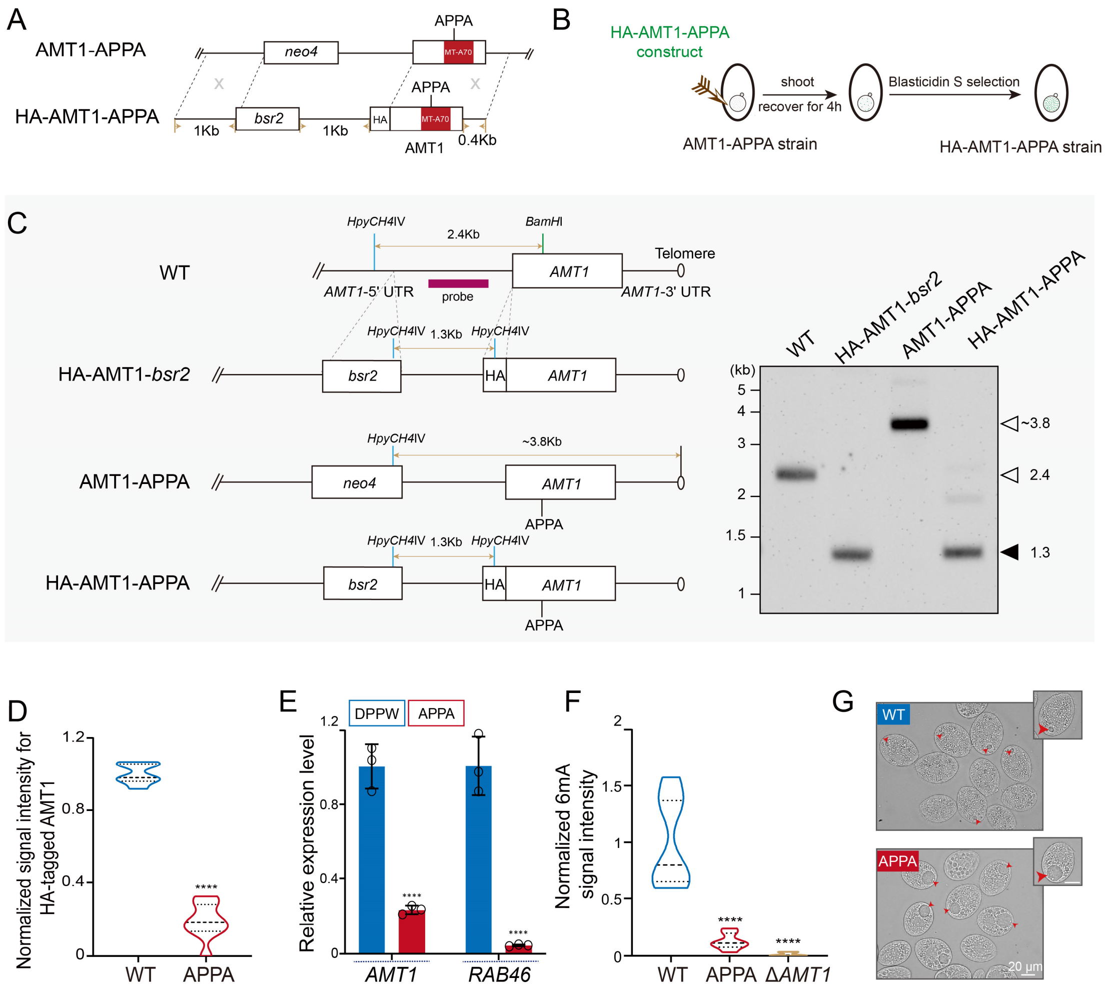

Fig. S2. Phenotypic analysis of AMT1-APPA cells.

A. Schematic representation of the endogenous *AMT1* locus in the MAC of AMT1-APPA cells and the HA-AMT1-APPA construct. The conserved DPPW motif was replaced by APPA.

B. Diagram illustrating the process of generating HA-AMT1-APPA cells. Starved AMT1-APPA cells were transformed with the HA-AMT1-APPA construct. True transformants were selected by increasing drug (blasticidin S) pressure.

C. Southern blot analysis showed that the endogenous *AMT1* locus was completely replaced in HA-AMT1-*bsr2* and HA-AMT1-APPA cells. gDNA was digested with *BamH*I and *HpyDH4*IV, and hybridized with probe generated from gDNA of wild-type (WT) amplified using primers AMT1-5f3931 and AMT1-5r4953. Both HA-AMT1-*bsr2* and HA-AMT1-APPA displayed a 1.3 kb band, indicating complete replacement. Unreplaced WT cells exhibited a 2.4 kb band, while AMT1-APPA cells displayed a 3.8 kb band.

D. Statistical analysis of IF signal intensity for HA-tagged AMT1 in HA-AMT1 (WT) and HA-AMT1-APPA (APPA) cells. Cell images were randomly selected (n (HA-AMT1-MAC) = 210 and n (HA-AMT1-APPA) = 241) by ImageJ. Data were presented as violin plots using GraphPad Prism 8. Student's *t*-test was performed (*****P* < 0.0001).

E. RT-qPCR analysis showed that expression levels of *AMT1* and *RAB46* genes were reduced in AMT1-APPA cells than those in WT cells. Expression level of *JMJ1* gene were as the internal control (Table S1). Three technique replicates were performed and averaged to represent the relative abundance. Student's *t*-test was performed (*****P* < 0.0001).

F. Statistical analysis of 6mA IF signal intensity in WT (SB210), AMT1-APPA, and Δ*AMT1* cells. Cells were randomly selected (n (WT) = 297, n (AMT1-APPA) = 477 and n (Δ*AMT1*) = 305)) by ImageJ. Data were presented as violin plots using GraphPad Prism 8. Student's *t*-test was performed (*****P* <0.0001).

G. Contractile vacuoles (CV) (red arrowheads) in WT and AMT1-APPA cells revealed that the CVs were significantly larger in AMT1-APPA cells.

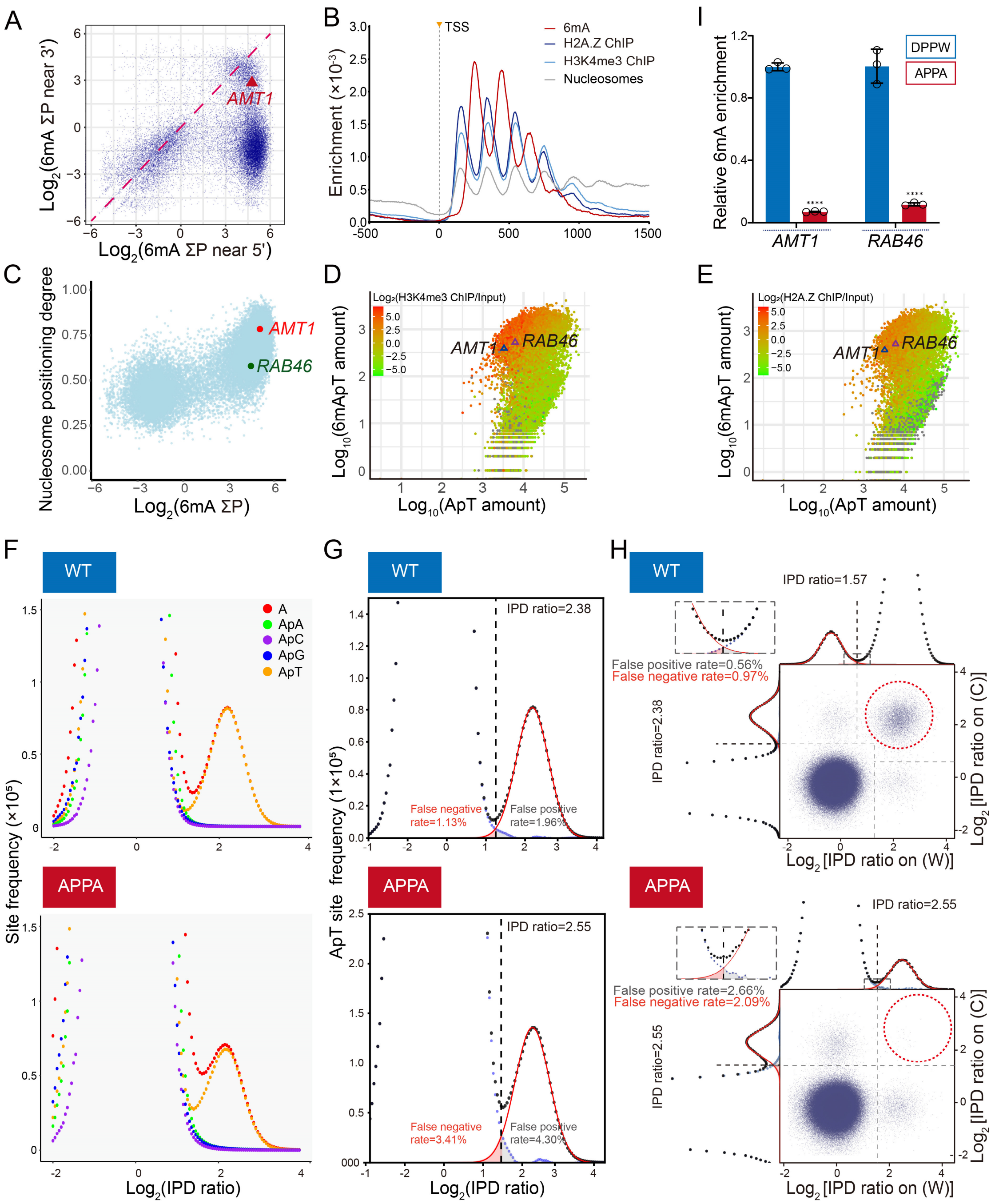

Fig. S3. SMRT-CCS data analysis in AMT1-APPA cells.

A. Preferential distribution of 6mA at the 5' end of the gene body. Each gene was equally divided into regions near the 5' or 3' ends based on the midpoint of its length. For each gene, the penetrance within the 5' and 3' region was calculated as 6mA ΣP near 5' and 6mA ΣP near 3', respectively. The red dashed line represented y = x, and the red triangle highlighted the *AMT1* gene.

B. Distribution profiles of 6mA, H2A.Z, H3K4me3, and nucleosome around 500 bp upstream and 1.5 kb downstream of the transcription start sites (TSSs).

C. The scatterplot, depicting the correlation between the nucleosome positioning degree and 6mA levels (ΣP) in each gene, revealed two clusters: the top right cluster had high 6mA levels and well-positioned nucleosomes, while the bottom left cluster

had relatively low 6mA levels and weakly positioned nucleosome. The *AMT1* and *RAB46* genes were located in the right cluster, exhibiting high 6mA levels and well-positioned nucleosomes.

D. The scatterplot depicting the correlation between H3K4me3 enrichment and 6mA levels. *AMT1* and *RAB46* were located in the top left corner in red, exhibiting high enrichment of 6mA and H3K4me3.

E. The scatterplots depicting the correlation between H2A.Z enrichment and 6mA levels. *AMT1* and *RAB46* were located in the top left corner in red, exhibiting high enrichment of 6mA and H2A.Z.

F. The plot depicting IPD ratio distributions of all A sites (ApA, ApC, ApG, and ApT dinucleotide) in WT (top) and AMT1-APPA cells (bottom). 6mA was exclusively present in the ApT dinucleotide.

G. The plot depicting the deconvolution of the 6mA peak and the unmodified A peak for IPD ratios distributions at the ApT dinucleotide. The curve of red line depicted the distribution peak of 6mApT based on Gaussian fitting (right). The curve of black dot represented the distribution peak of ApT (left) and the distribution peak of 6mApT (right) based on sequencing data. The residual between the sequencing data and the fitted value was showed by the curve of blue dot. In the screening criteria (WT cells: 6mA IPD ratio = 2.38, AMT1-APTA cells: 6mA IPD ratio = 2.55), integral area ratio severally represents the false positive rate (gray) and the false negative rate (red). Note the low false positive and false negative rates of 6mA calling in WT (top) and AMT1-APPA cells (bottom).

H. Demarcation of the four methylation states of ApT duplexes in WT (top) and AMT1-APPA cells (bottom) by their IPD ratios on Watson (W) and Crick (C) strands, respectively. Different IPD ratios thresholds (shifted according to the bimodal distribution using Python script) were used to determine the methylation status of each ApT dinucleotide. The ratio of hemi- and full-methylated 6mApT positions appeared to be pronouncedly different between AMT1-APPA and WT cells.

I. qPCR analysis of 6mA IP samples showed that 6mA levels of both *AMT1* and *RAB46* genes were reduced in AMT1-APPA cells compared to WT cells. Primers for unmethylated rDNA genes were used as the internal control (Table S1). Three technique replicates were performed and averaged to represent the relative abundance. Student's *t*-test was performed (*****P* < 0.001).

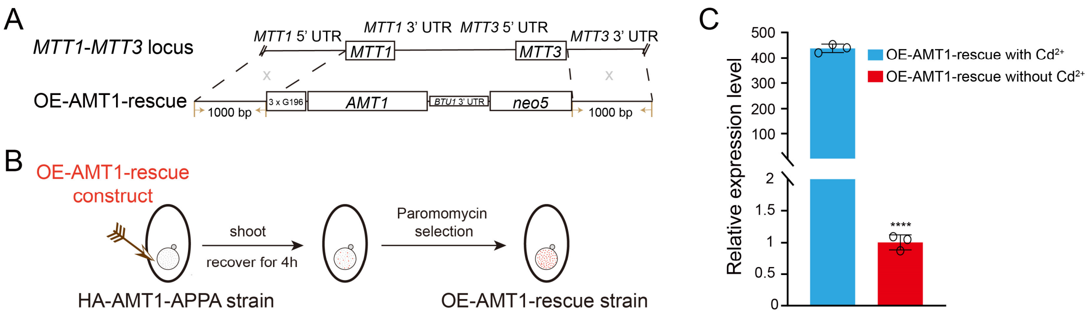

Fig. S4. Rescue of *AMT1* from an ectopic locus in AMT1-APPA cells.

A. Schematic representation of the *MTT1-MTT3* locus in the MAC of HA-AMT1-APPA cells and the OE-AMT1-rescue construct.

B. Diagram illustrating the process of generating OE-AMT1-rescue cells. Starved HA-AMT1-APPA cells were transformed with the OE-AMT1-rescue construct. True transformants were selected by increasing drug (paromomycin) pressure.

C. RT-qPCR analysis of mRNA showed that ectopic *AMT1* expression levels were significantly elevated in OE-AMT1-rescue cells induced with 1μg/mL Cd^{2+} for 17 h compared to that without Cd^{2+} induction using the primers of 3×G196-RT-f and AMT1-RT-r. *JMJ1* gene was as the internal control.

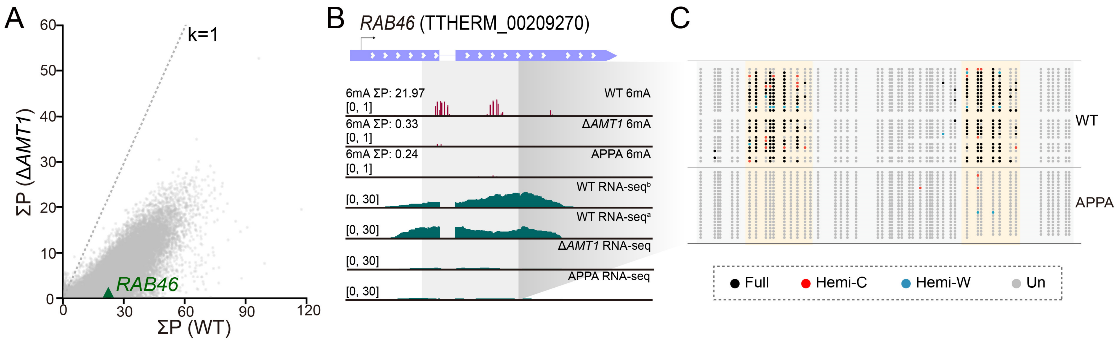

Fig. S5. The 6mA and mRNA levels of *RAB46* in AMT1-APPA cells.

A. 6mA levels of individual genes were dramatically reduced in Δ*AMT1* cells compared to WT cells. 6mA level of a specific gene was calculated as the sum of penetrance of all 6mApT positions in the gene body (ΣP). Note that the ΣP values 6mA level for the *RAB46* gene was located below the diagonal line (k=1), indicating a greater reduction in its 6mA level in Δ*AMT1* cells.

B. IGV snapshot of the *RAB46* gene locus. Tracks from top to bottom were: gene model, 6mA levels (SMRT-CCS data) of WT, Δ*AMT1* and AMT1-APPA cells, and mRNA levels (RNA-seq data) of WT, Δ*AMT1* and AMT1-APPA cells. Note that both 6mA levels and *RAB46* expression were reduced in Δ*AMT1* and AMT1-APPA cells. RNA-seq data from WT[a] was specifically used for the transcriptional comparison with AMT1-APPA, while RNA-seq data from WT[b] was used for the transcriptional comparison with Δ*AMT1*.

C. Single molecules covering the *RAB46* gene locus (highlighted in light gray in the IGV snapshot of Supplementary Figure S4E) in WT (top) and AMT1-APPA (bottom) cells. Notably, the 6mApT sites of the *RAB46* gene in AMT1-APPA cells were abolished compared to those in WT cells, particularly for fully methylated 6mApT.

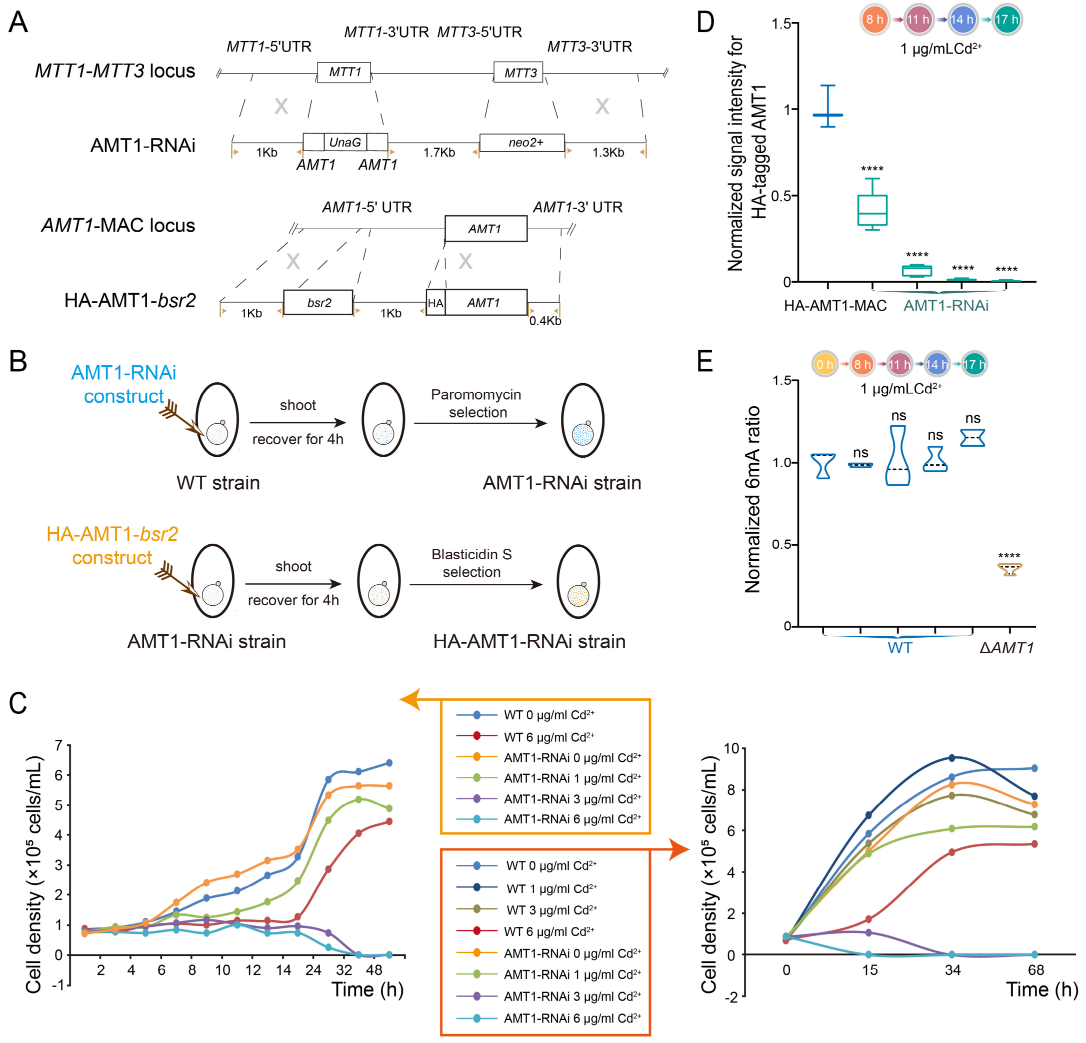

Fig. S6. Phenotypic analysis of AMT1-RNAi cells.

A. Schematic representation of the endogenous *MTT1-MTT3* locus in the MAC of WT cells and the *AMT1* knockdown (AMT1-RNAi) construct (top), and the endogenous *AMT1* locus in the MAC of AMT1-RNAi cells and the HA-AMT1-*bsr2* construct (bottom). The *MTT1* was replaced by the *UnaG* flanked with two short reversed complementary *AMT1* sequences. The *MTT3* was replaced by a drug cassette (*neo2+*).

B. Diagram illustrating the process of generating AMT1-RNAi (top) and HA-AMT1-RNAi cells (bottom). Starved WT and AMT1-RNAi cells were respectively transformed with the AMT1-RNAi and the somatic HA-AMT1-*bsr2* constructs. True transformants were selected by increasing drug (paromomycin or blasticidin S) pressure.

C. Growth rates of WT and AMT1-RNAi (with 0 μg/mL, 1 μg/mL, 3 μg/mL and 6 μg/mL Cd^{2+} treatment) cells. The number of cells were enumerated at the indicated time points using the Coulter counter. Doubling times (h) were calculated based on the log-phase data.

D. Statistical analysis of HA-tagged AMT1 IF signal intensity. Cell images were randomly selected (n (HA-AMT1-MAC) = 129, n (AMT1-RNAi-1 μg/mL-8 h) = 219,

n (AMT1-RNAi-1 μg/mL-11 h) = 192, n (AMT1-RNAi-1 μg/mL-14 h) = 191, n (AMT1-RNAi-1 μg/mL-17 h) = 225) by ImageJ. Data were presented as box plots. Student's *t*-test was performed (*****P* < 0.0001).

E. Mass spectrometry analysis of 6mA for three biological replicates in WT induced with 1μg/mL Cd^{2+} for 0h, 8 h, 11 h, 14 h, and 17 h, and Δ*AMT1* cells. Data were presented as violin plots. Student's *t*-test was performed (ns > 0.05).

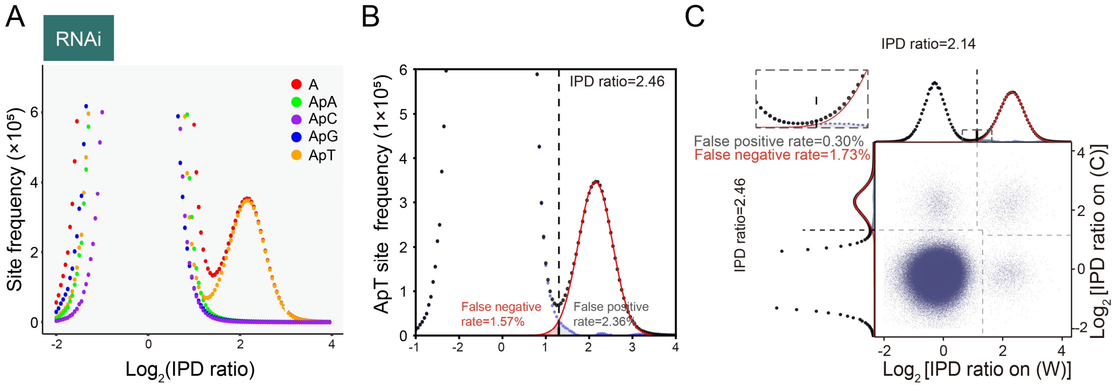

Fig. S7. SMRT-CCS data analysis in AMT1-RNAi cells induced by 1 μg/mL Cd^{2+} for 17 h.

A. The plot depicting IPD ratios distributions of all A sites (ApA, ApC, ApG, and ApT dinucleotide) in induced AMT1-RNAi cells. 6mA was exclusively present in the ApT dinucleotide.

B. The plot depicting the deconvolution of the 6mA peak and the unmodified A peak for IPD ratio distributions at the ApT dinucleotide in induced AMT1-RNAi cells. The curve of red line depicted the distribution peak of 6mApT based on Gaussian fitting (right). The curve of black dots represented the distribution peak of ApT (left) and 6mApT (right) based on sequencing data. The residual between the sequencing data and the fitted curve was shown by the curve of blue dots. Under this selection criteria (induced AMT1-RNAi cells: 6mA IPD ratio = 2.46), integral area ratio severally represents the false positive rate(gray) and the false negative rate (red).

C. Demarcation of the four methylation states of ApT duplexes in induced AMT1-RNAi cells by their IPD ratios on W and C strands, respectively. Different IPD ratios thresholds (shifted according to the bimodal distribution using Python script) were used to determine the methylation status of each ApT dinucleotide. The ratio of hemi- and full-methylated 6mApT positions appeared to be different between WT and induced AMT1-RNAi cells.

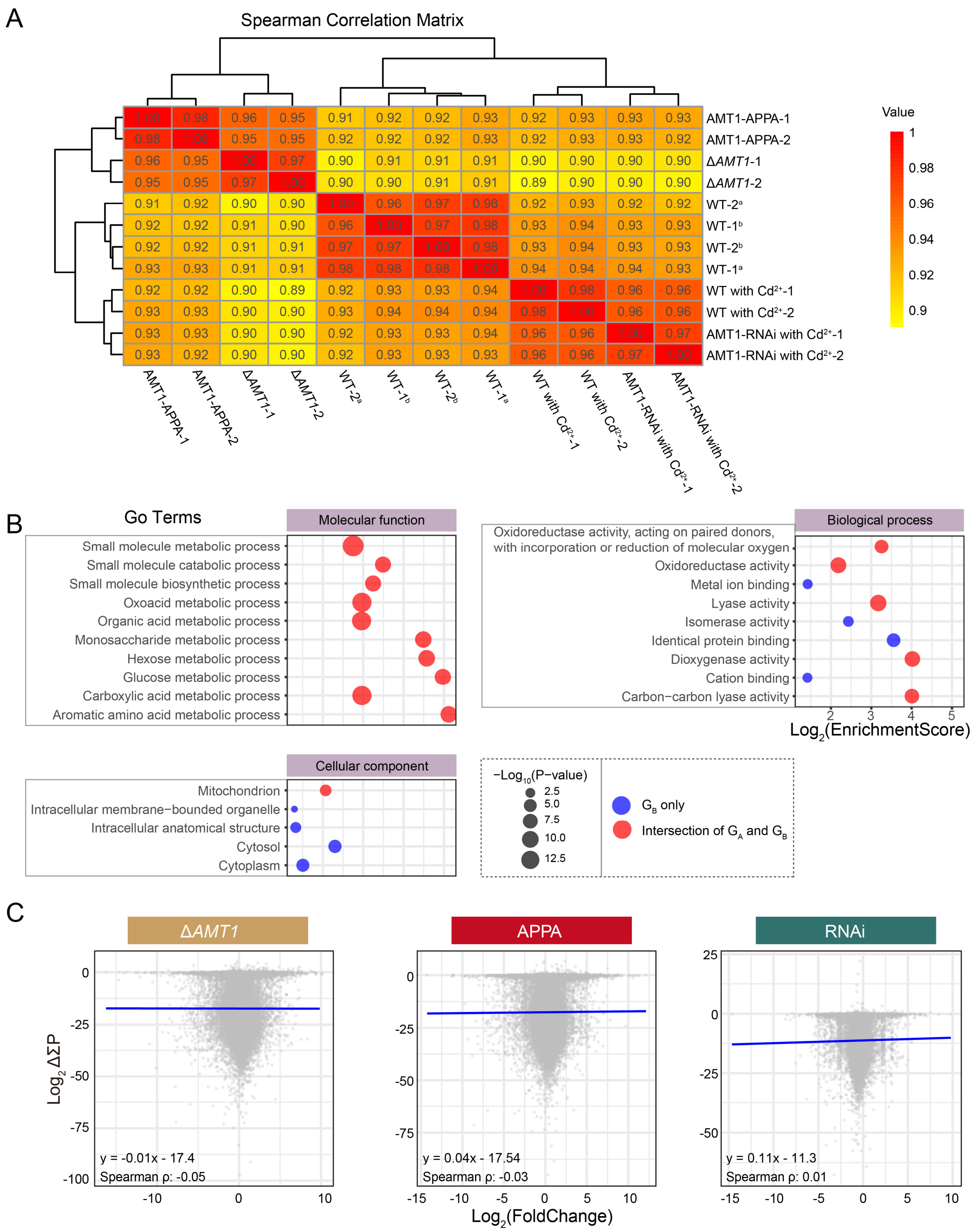

Fig. S8. Transcriptome analysis in the AMT1-APPA, AMT1-RNAi induced by 1 μg/mL Cd^{2+} for 17 h and Δ*AMT1* cells.

A. Hierarchically clustered correlation matrix of two replicates in WT, AMT1-APPA, Δ*AMT1*, Cd^{2+} induced WT and AMT1-RNAi (1 μg/mL Cd^{2+} for 17 h) cells. Spearman correlations were calculated.

B. Gene ontology term analysis on 141 genes that were co-downregulated in both 6mA and transcription levels among Δ*AMT1*, AMT1-APPA, and AMT1-RNAi (induced by 1μg/mL Cd^{2+} for 17 h) cells. The blue dot indicated pathways unique to Group B, while the red dot highlighted the intersected pathways between Group A

and Group B. The top 10 terms with the lowest p-values in each GO Class were displayed.

C. The scatterplots depicting the non-significant correlation between the changes of 6mA levels and mRNA levels among all genes in Δ*AMT1*, AMT1-APPA and AMT1-RNAi cells induced by 1 μg/mL Cd^{2+} for 17 h.

Supplemental tables S1-S6.

Table S1. Primers used for construct generation.

Primer	Sequences (5'-3')	Notes
AMT1-5f1	CGGCCGCCACCGCGGTGGAGCTCGTGGTCCAATATTATTGGCTCTAAC	For making HA-AMT1-MAC construct
AMT1-5r1	CAAAACCCGTCAGGTGCCTGGTAGTCTAACAGATTTATGTTCTGC	
AMT1-5f2	GGAAAAACTGACGTCGCACCATGCAAAGTTGAGAGATTATAGCAATAAG	
AMT1-3r1	CTAAAGGGAACAAAAGCTGGAGCTCGATTAATCTCTGATTAACTTAC	
HA-AMT1-f4198	TACCCCTACGACGTTCCCGACTACGCCTCAAAAGCAGTTAATAAAAAG	
HA-AMT1-r4197	GGCGTAGTCGGGAACGTCGTAGGGGTACATTTTGAACTTGATAATTATA	
Bsr2-f1	CGCTCTAGAACTAGTGGATCCCCCGGGTAACAGGCACCTGACAGCTGC	For making HA-AMT1-*bsr2* construct
Bsr2-r1	CTTGATATCGAATTCCTGCAGCCCGGGATGGTGCGACGTCAGTTTTTC	
AMT1-f4815-D265AW268A	GATGGCTCCTCCTGCGTAACTCTCATCCTCTCAACCTTCAAGAGGTG	For making HA-AMT1-APPA construct
AMT1-r4836-D265AW268A	GAGTTACGCAGGAGGAGCCATCATAATAACATCGAACATCTTACCAG	
AMT1-RNAi-f	GGTAGTGGATCAGGCGCGTGAATAAAGTGATGAAGATTACG	For making AMT1-RNAi construct
AMT1-RNAi-r	GACGTCGTAGGGGTATCCGTGAATAAAGTGATGAAGATTACG	
AMT1-AscI-r	CCACCTCACTAGGCGCGCCGTAGTCATCCTCTTATGTAGAGGC	
AMT1-AscI-f	CCACCAACTTTTCTTGATCCTGCAGGTAGTCATCCTCTTATGTAGAGGC	
AMT1-MIC-5f210-SacI-infusion	CTAAAGGGAACAAAAGCTGGAGCTCCTAGCTGTTTTCTTGTCAATCC	For making germline HA-AMT1 construct
AMT1-MIC-5r1879-NotI-infusion	GATCCACTAGTTCTAGAGCGGCCGCCAATATTTTTGGAACAGGGA	
AMT1-MIC-5f1889-infusion	CTTATCGATACCGTCGACCTCGACAATAGAAGCTATTTTATATG	
AMT1-MIC-r3526-XhoI-infusion	GAATTGGGTACCGGGCCCCCCCTCGAGCATAATTCATTTCCTATTGAAACC	
AMT1-qPCR-5f2415	GATATGCCACTATCAGAGATAGG	For qPCR of HA-AMT1-MAC and
AMT1-qPCR-5r2649	CTTAGTTGCAATTAAATAGAATC	

JMJ1- f2071	CTATCTAACGGAGTAATGTTTGCTG	HA-AMT1-RNAi
JMJ1-r2236	AAGGTTGAGTGCATCCCATAACG	
neo4-f883	TGAACAAGATGGTTTACACGCTGG	For qPCR of HA-AMT1-APPA
neo4-r1057	GACGGGCAGCTTCATCTTGAAGTTC	
AMT1-5f3931	GAAATTTAAGAACAATAATTC	For Southern blot of AMT1 locus
AMT1-5r4953	GCAATAATTAAACTTTTCTTG	
AMT1-f1054	GAATAAAATGAAATAGCTAATGG	For 6mA-IP qPCR of AMT1-APPA strain
AMT1-r1185	CGAACATCTTACCAGCGTGTCTC	
RAB46-f328	CATTACTAATAGATACTCCTTTG	
RAB46-r404	CTACTTTATTTAATTTTTTTTAATTG	
rDNA-f1038	GTAAATCTTTTGTAGACGACTTAAC	
rDNA-r1106	GATCGTAGGAAATTCTACTCTCATGC	
RAB46-RTqPCR-f2018	GTAGTATAAAATTATGAAAGTAGC	For RT-qPCR of AMT1-APPA strain
RAB46-RTqPCR-r2246	CAAAATTAAAATTTAATTTGTTC	
AMT1-RTqPCR-f4905	CATGCCAATCCAATCTCTATAACAAG	
AMT1-RTqPCR-r5155	CTTTCTTTAGCATGTTGCAAGTAG	
JMJ1-RTqPCR-f2244	CCTAAAAGTCGCTGGAGTGTGG	
JMJ1-RTqPCR-r3176	CCATAGACCTTCATCGCCATG	
OE-3×G196-AMT1-FW	GAAATGCTGACTTAGTTCCTAGAGGATCCATGTCAAAAGCAGTTAATAAAAAGGG	For making OE-AMT1-rescue construct
OE-3×G196-AMT1-RV	GCTGACCGATTCAGTTCGCTCAACTAGTTCATAATTCATTTCCTATTGAAACCC	
3×G196-RT-f	GTTCCTAGAGGAAATGCTGAC	For RT-qPCR and 6mA-IP qPCR of OE-AMT1-rescue strain
HA-RT-f	CCCTACGACGTTCCCGACTACG	
AMT1-RT-r	GCACTTTTTTTATTATTTGCTG	

Table S2. Strains used in this study.

Strain	Manipulation	Drug cassette	Somatic (S) or Germline (G)	Background	Mating type	Source
HA-AMT1-complete	HA tag	neo4	G	SB210 & CU428	II	This study
HA-AMT1-complete	HA tag	neo4	G	SB210 & CU428	VI	This study
HA-AMT1-MIC	HA tag	neo4	G*	SB210 & CU428	VI	This study
HA-AMT1-MIC	HA tag	neo4	G*	SB210 & CU428	VII	This study
HA-AMT1-MAC	HA tag	neo4	S	SB210	VI	This study
HA-AMT1-MAC	HA tag	neo4	S	CU428	VII	This study
HA-AMT1-*bsr2*	HA tag	bsr2	S	SB210	VI	This study
Δ*AMT1*-complete	Knockout	neo4	G	SB210 & CU428	II	This study
Δ*AMT1*-complete	Knockout	neo4	G	SB210 & CU428	V	Wang et al., 2019
Δ*AMT1*-MIC	Knockout	neo4	G*	SB210 & CU428	IV	This study
Δ*AMT1*-MIC	Knockout	neo4	G*	SB210 & CU428	VI	This study
AMT1-RNAi	Knockdown	neo2+	S	SB210	VI	This study
HA-AMT1-RNAi	HA tag	bsr2/neo2+	S	AMT1-RNAi	VI	This study
AMT1-APPA	Point mutant	neo4	G	SB210 & CU428	II	Wang et al., 2019
HA-AMT1-APPA	HA tag	bsr2/neo4	S	AMT1-APPA	II	This study

OE-AMT1-rescue	3×G196 tag	neo5	S	HA-AMT1-APPA	II	This study

Somatic (S): The targeted construct was transformed into the macronuclei.

Germline (G): The targeted construct was transformed into both macronuclei and micronuclei.

G*: The targeted construct was transformed only into the micronuclei.

Table S3. Sexual progeny viability in WT, Δ*AMT1*-complete, and Δ*AMT1*-MIC cells.

Strains	Percentage of viable sexual progeny	Number of mating pairs	Number of viable sexual progeny
WT	88.2 %	144	127
Δ*AMT1*-complete	4.9 %	144	7
Δ*AMT1*-MIC	16.7 %	144	24

Table S4. Quality control of SMRT-CCS.

Job Metric	WT	AMT1-APPA	AMT1-RNAi	Δ*AMT1*
Number of bases	427,557,740,933	211,589,891,986	410,702,940,636	416,917,400,924
Number of single molecules	3,859,218	1,384,963	3,488,821	5,820,416
Max polymerase read length	464,740	444,582	602,905	420,914
N50 polymerase read length	210,155	257,519	218,647	163,048
Mean polymerase read length	110,788	152,776	117,720	71,630
Percentage of short fragment length (<500bp, %)	1.73	0.15	2.86	2.75
Median fragment length	2,630	4,126	2,395	3,345
Number of N* clusters	126,727	128,597	171,298	60,041
Number of single molecules with global dispersion of IPD ratio	51,594	82,217	110,088	29,727
Number of single molecules used for downstream analysis	1,245,340	556,599	1,126,609	762,624
Number of detected ApT sites at the ensemble level	27,804,246 (99.90%)	27,756,302 (99.73%)	27,828,464 (99.99%)	27,102,680 (97.38%)
Average coverage (×)	30	21.5	24.5	28

Table S5. 6mA statistics of SMRT-CCS in WT, AMT1-APPA, and AMT1-RNAi cells with 1 µg/mL Cd^{2+} treatment for 17h.

Single molecule	WT		AMT1-APPA		AMT1-RNAi		*ΔAMT1*	
	Number	Percentage (%)	Number	Percentage (%)	Number	Percentage (%)	Number	Percentage (%)
A sites	2,430,618,393	-	1,735,206,462	-	1,964,123,883	-	1,730,640,574	-
6mA sites	17,889,360	0.74	3,552,315	0.20	7,471,566	0.38	3,443,531	0.20
ApT sites	823,903,428	-	597,310,764	-	681,211,300	-	645,000,722	-
6mApT sites	16,765,614	2.03	2,975,184	0.50	6,948,007	1.02	3,420,511	0.53
Full	14,811,496	88.35	67,302	2.26	3,482,226	50.11	82,362	2.40
Hemi-C	974,677	5.81	1,457,879	49.00	1,733,326	24.95	1,670,284	48.83
Hemi-W	979,261	5.84	1,450,003	48.74	1,732,455	24.94	1,667,685	48.77
Total 6mApT	16,765,614	-	2,975,184	-	6,948,007	-	3,420,511	-

Table S6. Summary of differential expression analysis from RNA-seq, gene methylation levels from SMRT-seq, and GO enrichment analysis results for selected genes across different strains.

Table S6 is provided in Excel file.

Supplementary Materials and Methods.

Generation of *Tetrahymena* strains

Δ*AMT1* and AMT1-APPA strains were described previously (1). To generate the HA-AMT1-MAC construct, we introduced the hemagglutinin (HA) sequence to the 5' end of the endogenous *AMT1* gene coding region by cloning *AMT1* ORF and its flanking regions, and inserted the *neo4* cassette into *AMT1* 5' UTR (Supplementary Figure S1A-B). The 5' flanking sequences of *AMT1* upstream of the *neo4* cassette were amplified using the primers AMT1-5f1 and AMT1-5r1, and the sequences downstream of the *neo4* cassette was amplified using the primers of AMT1-5f2/3r1 (Table S1). The HA-AMT1-MAC construct was transformed into the MAC of WT cells to obtain the HA-AMT1-MAC strain (MAC: tagged by HA, MIC: WT).

For making the germline HA-AMT1 construct, primers of AMT1-MIC-5f210-SacI-infusion/AMT1-MIC-5r1879-NotI-infusion and AMT1-MIC-5f1889-infusion/AMT1-MIC-r3526-XhoI-infusion were used to amplify the 5' flanking sequences of *AMT1* upstream of the *neo4* cassette and the sequences downstream of the *neo4* cassette, respectively (Table S1). Germline strains (HA-AMT1-MIC and HA-AMT1-complete) were generated using the germline transformation with standard procedures (2,3). WT cells with two different mating types (SB210 and CU428) were mated after starvation for 16-18 h, and the germline HA-AMT1 construct was transformed into their MIC at 3h post-mixing. HA-AMT1 homozygous heterokaryon (HA-AMT1-MIC, MAC: WT, MIC: tagged by HA) was obtained by crossing the heterozygous heterokaryon HA-AMT1 strain with star strain (B*VI or B*VII). HA-AMT1 homozygous homokaryon (HA-AMT1-complete, MAC: AMT1 tagged by HA, MIC: tagged by HA) was obtained by crossing two homozygous heterokaryon strains.

For the HA-AMT1-APPA construct, APPA mutation was introduced into the HA-AMT1-*bsr2* construct using the primers AMT1_f4815_D265AW268A and AMT1_r4836_D265AW268A (Supplementary Figure S2A-B, Table S1). The HA-AMT1-APPA construct was transformed into the MAC of AMT1-APPA cells using the standard somatic transformation strategy (4).

For the AMT1-RNAi construct, two identical fragments containing 295 bp *AMT1* CDS were amplified using the primers AMT1-RNAi-f/AMT1-RNAi-r (Product-1) and the primers AMT1-*Asc*I-r/AMT1-*Asc*I-f (Product-2). Approximately 100 bp of the *UnaG* gene, 3' UTR of *MTT1*, 5' UTR of *MTT3*, the *neo2+* cassette, and 3' UTR of *MTT3* in the pBlueScriptSK(-) vector (1), were inserted downstream of the cadmium-inducible *MTT1* promoter, as well as Product-1 and Product-2 in opposite orientations, by T4 ligation at the *Sbf*I and *Asc*I sites, respectively. The *UnaG* gene was used as a spacer to produce hairpin RNA (5,6). The AMT1-RNAi construct was transformed into the MAC of WT cells to obtain the AMT1-RNAi strain. The

HA-AMT1-*bsr2* construct was transformed into the MAC of AMT1-RNAi cells (4,5) and selected by increasing the concentration of blasticidin S up to 1.6 mg/mL in SPP medium to obtain the HA-AMT1-RNAi strain (Supplementary Figure 6A-B). The replacement was analyzed by qPCR using primers AMT1-qPCR-f2415/r2649 for HA-AMT1-MAC and HA-AMT1-RNAi, and neo4-f883/r1057 for HA-AMT1-APPA, and by PCR using primers AMT1-f3665/r4242 for HA-AMT1-complete. The primers of JMJ1-f2071/r2236 were used for the internal control and normalization.

For HA-AMT1-*bsr2* and HA-AMT1-APPA strains, the replacement was also analyzed by Southern blot using AlkPhos Direct Labeling Module (Cytiva, RPN3680) (Supplementary Figure 2C) (7). In brief, genomic DNA from transformants was digested with *BamH*I and *HpyDH4*IV, and hybridized with the probe generated from DNA amplified with primers AMT1-5f3931 and AMT1-5r4953. The signal was generated by CDP-star (Cytiva, RPN3682) and detected by ChemiDoc Touch MP imaging system (Bio-Rad).

For OE-AMT1-rescue construct, the primers OE-3×G196-AMT1-FW and OE-3×G196-AMT1-RV were used to generate the *AMT1* fragment fused with a 3×G196 tag (Supplementary Figure 4A-B, Table S1). The *AMT1* fragment fused with a 3×G196 tag was then inserted downstream of the *MTT1* promoter followed by the *neo5* cassette. The OE-AMT1-rescue construct was transformed into HA-AMT1-APPA strains to generate OE-AMT1-rescue strain. All strains are listed in Table S2.

6mA IP

Genomic DNA was fragmented into 200-400 bp by sonication using Bioruptor Plus (Digenode). The fragmented DNA was denatured at 95 °C and chilled for 10 minutes on ice. A portion of DNA (1~10 μL) was used as the input sample. The rest of the DNA was incubated with anti-6mA antibody (Synaptic Systems, 202003, 1:200) in 500 μL 1×IP buffer (50 mM Tris-HCl pH 7.4, 750 mM NaCl, 0.5% Trion X-100 and 20 mM EDTA) at 4 °C overnight. Protein A magnetic beads (Thermo Fisher Scientific, 10001D) were washed three times in 1 mL 1×IP buffer, added to the DNA-6mA antibody mixture, and incubated with gentle rotation at 4 °C for 2 h. Beads were then washed three times with 1 mL 1×IP buffer. DNA was eluted twice by 100 μL of elution buffer (5 mM Tris-HCl pH 8.0, 0.5% SDS, 9% 1×IP buffer and 15% with 2.5 mg/mL proteinase K) at 4 °C for 1 h, which was precipitated by 20 μL of 3 M NaOAc pH 5.5, 500 μL of 100% EtOH, and 0.5 μL of 20 mg/mL glycogen at -80 °C overnight. The precipitated DNA was obtained by centrifuging at 14,000 g for 20 min at 4 °C.

References

1. Wang, Y., Sheng, Y., Liu, Y., Zhang, W., Cheng, T., Duan, L., Pan, B., Qiao, Y., Liu, Y. and Gao, S. (2019) A distinct class of eukaryotic MT-A70 methyltransferases maintain symmetric DNA N^6-adenine methylation at the ApT dinucleotides as an epigenetic mark associated with transcription. *Nucleic Acids Res.*, **47**, 11771-11789.
2. Cassidy Hanley, D., Bowen, J., Lee, J.H., Cole, E., VerPlank, L.A., Gaertig, J., Gorovsky, M.A. and Bruns, P.J. (1997) Germline and somatic transformation of mating *Tetrahymena thermophila* by particle bombardment. *Genetics*, **146**, 135-147.
3. Hai, B. and Gorovsky, M.A. (1997) Germ-line knockout heterokaryons of an essential α-tubulin gene enable high-frequency gene replacement and a test of gene transfer from somatic to germ-line nuclei in *Tetrahymena thermophila*. *Proc. Natl. Acad. Sci.*, **94**, 1310-1315.
4. Talsky, K.B. and Collins, K. (2012) Strand-asymmetric endogenous *Tetrahymena* small RNA production requires a previously uncharacterized uridylyltransferase protein partner. *RNA*, **18**, 1553-1562.
5. Howard-Till, R.A. and Yao, M. (2006) Induction of gene silencing by hairpin RNA expression in *Tetrahymena thermophila* reveals a second small RNA pathway. *Mol. Cell. Biol.*, **26**, 8731-8742.
6. Zhao, X., Xiong, J., Mao, F., Sheng, Y., Chen, X., Feng, L., Dui, W., Yang, W., Kapusta, A., Feschotte, C. *et al.* (2019) RNAi-dependent Polycomb repression controls transposable elements in *Tetrahymena*. *Genes Dev.*, **33**, 348-364.
7. Kataoka, K. and Mochizuki, K. (2015) Phosphorylation of an HP1-like protein regulates heterochromatin body assembly for DNA elimination. *Dev. Cell*, **35**, 775-788.
